# The relevance of body composition assessment for the rating of perceived exertion in trained and untrained women and men

**DOI:** 10.3389/fphys.2023.1188802

**Published:** 2023-08-01

**Authors:** Julia Lichti, Martina Anna Maggioni, Björn Balcerek, Philipp Nils Becker, Robert Labes, Hanns-Christian Gunga, Michael Fähling, Mathias Steinach

**Affiliations:** ^1^ Charité—Universitätsmedizin Berlin, Institute of Translational Physiology, Berlin, Germany; ^2^ Charité—Universitätsmedizin Berlin, Institute of Physiology, Center for Space Medicine and Extreme Environments Berlin, Berlin, Germany; ^3^ Department of Biomedical Sciences for Health, Università Degli Studi di Milano, Milan, Italy

**Keywords:** body composition, Borg scale, Bruce protocol, endurance, exercise test, exertion, training status, sex

## Abstract

**Introduction:** Mechanic power output (MPO) and oxygen consumption (VO_2_) reflect endurance capacity and are often stated relative to body mass (BM) but less often per skeletal muscle mass (SMM). Rating of perceived exertion (RPE) has previously shown conflicting results between sexes at submaximal intensities. Individual body composition, however, largely differs due to sex and training status. It was the aim of this study to evaluate RPE of untrained and trained individuals of both sexes considering body composition and to estimate whether RPE could be improved as a tool to determine endurance capacity.

**Methods:** The study included 34 untrained adults (age 26.18 ± 6.34 years, 18 women) and 29 endurance trained (age 27.86 ± 5.19, 14 women) who were measured for body composition (InBody 770, InBody Europe B.V., Germany) and tested on a treadmill (Pulsar, H/P/Cosmos, Germany) for aerobic capacity (Metalyzer 3B, Cortex Biophysik GmbH, Germany) in an all-out exercise test applying the Bruce-protocol. VO_2_, MPO, heart rate (HR), and RPE were obtained at each exercise stage. VO_2_ and MPO were calculated per BM and SMM. RPE values were correlated with absolute VO_2_ and MPO, as well as relative to BM, and SMM. HR values and the parameters’ standardized values served for comparison to standard procedures.

**Results:** VO_2_ and MPO were higher in men compared to women and in trained compared to untrained participants. No differences between groups and sexes exist when VO_2_ and MPO were calculated per BM. When calculated per SMM, VO_2_ and MPO indicate opposite results already at low intensity stages of exercise test. RPE values had highest correlation with MPO per SMM (R^2^ = 0.8345) compared to absolute MPO (R^2^ = 0.7609), or MPO per BM (R^2^ = 0.8176). Agreement between RPE and MPO per SMM was greater than between RPE and HR (*p* = 0.008).

**Conclusion:** Although RPE represents a subjective value at first glance, it was shown that RPE constitutes a valuable tool to estimate endurance capacity, which can be further enhanced if individual body composition is considered. Furthermore, MPO and VO_2_ should be considered relative to SMM. These findings might help to avoid over-exertion, especially among untrained people, by adjusting the training intensity for each subject according to the individual strain evaluated in an exercise test based on individual body composition.

## Highlights

### Key points summary

• Rating of perceived exertion, when based on sex and body composition, is more precise and informative, than expressed in absolute values or referring to simply body mass.
• Rating of perceived exertion, based on sex and body composition, may be a crucial tool for training monitoring and guidelines development.
• Rating of perceived exertion, as a differentiating tool based on sex and body composition, performs best at submaximal exercise levels.

## Introduction

Measurements of physical work capacity using cycle ergometers and treadmills have been widely used to assess human endurance training status (Mazaheri et al., 2021; Wiecha et al., 2022). Commonly obtained parameters are achieved mechanic power output (MPO) to compare raw physical performance (Cavagna and Kaneko, 1977; Samozino et al., 2016) and maximal oxygen consumption (VO_2_max) to compare aerobic capacity (Levine, 2008; Wang et al., 2010) among patients (Mazaheri et al., 2021), recreational athletes (Shephard, 2009), and highly trained professionals (Podlogar et al., 1985).

VO_2_ and VO_2_max values are commonly reported per body mass, but less commonly per lean body mass, fat free mass, or skeletal muscle mass (SMM), in order to offset sex differences based on varying body composition between the sexes (Lewis et al., 1986; Price et al., 2022). Nevertheless, men may still yield higher VO_2_max values of up to 27% based on fat free mass compared to women (Davis et al., 2006). Since VO_2_ resembles energy expenditure of about 20 kJ/l oxygen (Brooks, 2012) and is connected to MPO through the factor of exercise efficiency (Böning et al., 2017), evaluating MPO not only in absolute terms or per body mass (BM) (Hettinger et al., 1961), but also per SMM would be of interest. However, this information is lacking in current literature. As sex differences mainly present themselves in variable percentages of muscle mass, with higher muscle mass among men of about 38% body mass compared to 31% body mass among women across all age groups (Janssen et al., 2000), strong influences on individual exercise performance may be assumed. Nonetheless, only few information exist that evaluate power output with regard to body composition (Gulmans et al., 1997; Cavalheri et al., 2010). One of these studies showed an influence of sex for maximal attained MPO per fat free mass among children with greater values attained by boys of 5.26 W/kg fat free mass compared to 5.06 W/kg fat free mass by girls (Gulmans et al., 1997). The other study found that maximal MPO among COPD patients is better estimated by the product of a 6 min walking test and fat free mass (correlation coefficient R = 0.64), than by a previously proposed equation, which based merely on body mass (R = 0.54) (Cavalheri et al., 2010). However, these studies evaluated children and COPD patients, respectively, but not healthy adults and did not consider the influence of sex and endurance training status.

The Borg scale, or rating of perceived exertion (RPE), represents a widely applied tool to obtain individual perceived exertion in various settings (Borg, 1970; Lollgen et al., 1980; Boxman-Zeevi et al., 2022). Both parameters have been shown to correlate with other indicators of physical strain such as heart rate (HR), relative VO_2_, feelings of pain, respiratory fatigue, and reaching onset of blood lactate accumulation (Borg et al., 1985; Scherr et al., 2013). Especially, the 15-grade scale (ranging from 6 to 20) was “constructed to give a fairly linear increase with heart rate” (Borg, 1998) in order to link subjectively rated exertion with a measurable physiological parameter indicative of exercise strain. However, few attempts have been made to analyze different responses of perceived exertion based on sex with conflicting results. According to the data gathered hitherto, women rated less intense in naturally occurring leg muscle pain at peak power output with 5.5 ± 2.9 compared to men with 8.3 ± 2.3 on a peak pain scale of 1–10 (Cook et al., 1998). In contrast, women reported significantly higher dyspnea ratings at an exercise intensity of 2.0 l VO_2_ and at 6 METs with dyspnea ratings of 4.0 ± 0.3 and 3.7 ± 0.4, respectively, compared to male participants with dyspnea ratings of 2.8 ± 0.3 and 2.6 ± 0.2, respectively (Phillips et al., 2019).

So far, no attempt has been made to evaluate the influence of sex and training status on individual RPE and its relation to objectively measurable physical strain parameters such as MPO and VO_2_ based on individual body composition at different exercise intensities. It was the aim of this study to fill this gap to gain better insight on how healthy adults of different sex and training status perceive physical strain. Moreover, the study aimed at evaluating whether VO_2_ and MPO based on individual SMM will provide additional knowledge in determining RPE. Such analysis may offer an individualized recommendation of training intensities based on individual body composition. To address these aims, three hypotheses were constructed: 1) Objectively measurable parameters of physical strain will differ between the sexes and training status dependent on their representation either as absolute values, or relative to body mass or SMM; 2) Individually reported RPE show a greater correlation to VO_2_ and MPO when they are calculated per SMM; 3) Compared to the agreement between RPE and HR, the agreement between RPE and VO_2_ and MPO relative to SMM will add additional knowledge.

## Methods

### Subjects and study preparation

**FIGURE 1:**
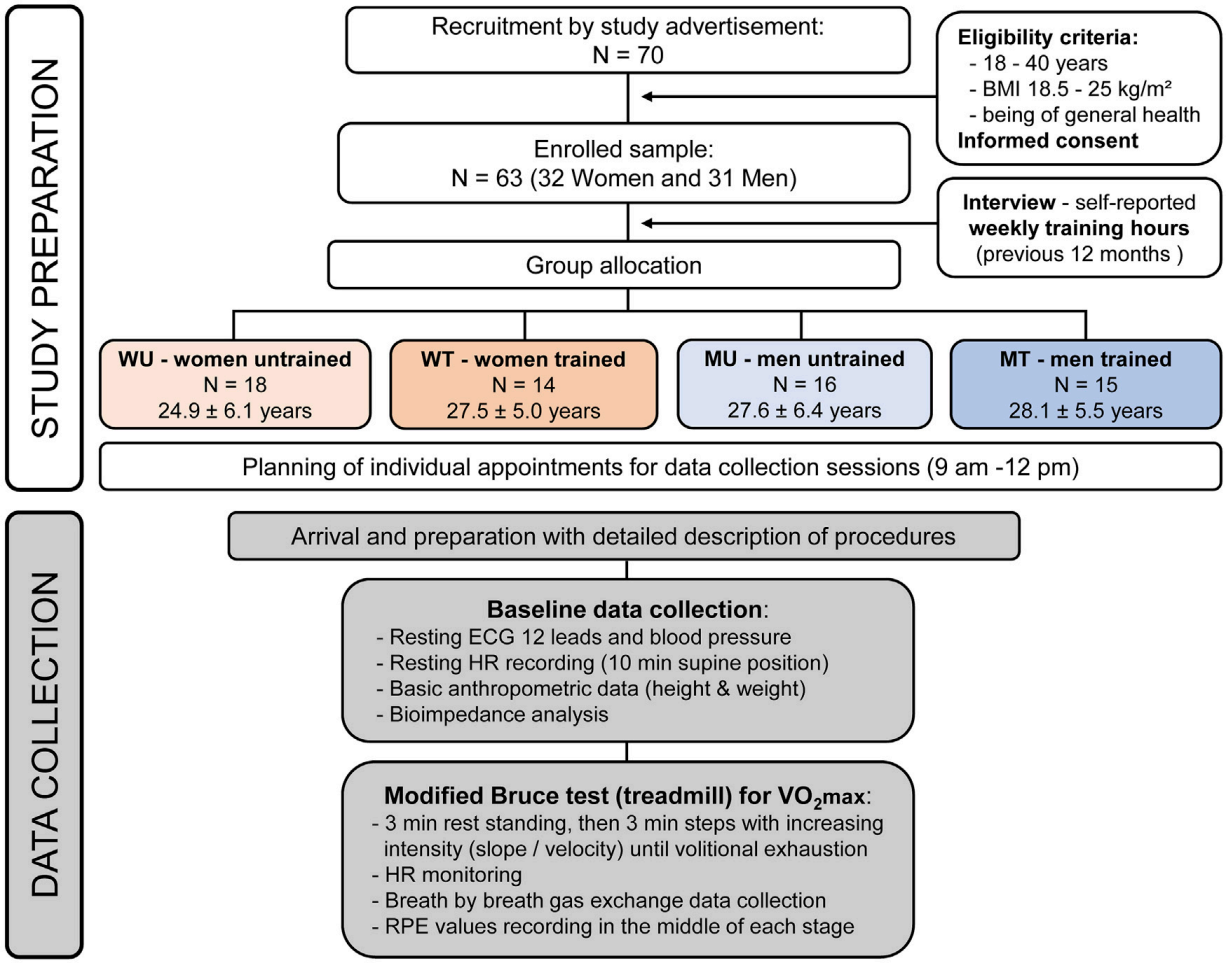
Visualization of the recruitment and measurement process within the study.

A number of n = 63 adults were recruited as part of a larger investigation through online and in-print advertisements briefly outlining the study as described in previous reports (Balcerek et al., 2020; Rabuffetti et al., 2021). Inclusion criteria were age (18–40 years), BMI (18.5–25 kg/m^2^), endurance training status (either being endurance trained or not) and being of general and cardiac health (including an asymptomatic electrocardiogram at rest). Based on their biological sex (either being a woman or a man, W or M) and based on self-reported weekly training hours, as described below, participants were divided in four groups: women untrained (WU, 18 subjects, mean age 24.94 ± 6.16 years), women trained (WT, 14 subjects, mean age 27.57 ± 5.02 years), men untrained (MU, 16 subjects, mean age 27.56 ± 6.44 years), and men trained (MT, 15 subjects, mean age 28.13 ± 5.51 years). Classified as trained (T) were participants who exercised at least three times per week for a minimum of 1 h per session for at least 1 year prior to the study, whereas subjects who did none or just occasional exercise were classified as untrained (UT). The recruitment process is indicated in Figure 1 (upper part).

The potential study participants had several weeks to pose questions and to decide to partake in the study. The study was approved by the Charité Ethics Board (IRB-number: EA1/154/18). All conducted measurements and procedures complied with the Declaration of Helsinki (7th revised version, 64th World Medical Association meeting, Fortaleza, Brazil) concerning the treatment of human subjects. All participants gave their informed written consent.

### Experimental protocol during data collection sessions

All tests were performed from 9 a.m. to 12 p.m. under standardized conditions (19°C–22°C ambient temperature, 99–102 kPa ambient air pressure, and 40%–50% relative ambient humidity) between February and April 2019. Participants gave information about their training and received a description of the test procedures. Blood pressure and electrocardiogram (ECG) were measured from each subject and evaluated by a medical doctor along with a short medical anamnesis to exclude contraindications for a maximal exercise test. Resting heart rate (RHR) were obtained through a mobile HR monitor (RS800CX, Polar Electro Oy, Kempele, Finland) that has been validated for scientific use (Hernando et al., 2018), while participants remained in a supine position for 10 min. BM and height were measured using a calibrated scale (Seca GmbH, Hamburg, Germany) and the BM index (BMI) was calculated from these values. Furthermore, body composition parameters Fat Mass (FM) and SMM were obtained using a body composition analyzing device (InBody770, InBody Europe B.V., Eschborn, Germany), validated for scientific use (McLester et al., 2020), as described in a previous work (Balcerek et al., 2020). All anthropometric measurements were performed after voiding the participants’ bladder and while wearing only light sportswear.

Subsequently, an exercise test was carried out on a treadmill (Pulsar, H/P/Cosmos, Nussdorf-Traunstein, Germany) that has been validated for scientific application (Hottenrott KS and Hübel, 2005), using the Bruce protocol (Bruce et al., 1963) until maximum exhaustion. This protocol is a reliable means to determine the VO_2_max defined as the maximum amount of oxygen to be utilized (Hall-Lopez et al., 2015). Continuous measurements of VO_2_ were taken using a breath-by-breath gas analysis system (Metalyzer 3B, Cortex Biophysik GmbH, Leipzig, Germany), which has been validated for scientific use (Meyer et al., 2001). Velocity and slope were gradually increased with each stage (Supplementary Table S1). Again, the same HR monitors were used to obtain HR during the exercise test. To reach their maximum performance, participants were verbally encouraged to carry out the test as long as possible (Midgley et al., 2018). In addition, further objective maximal test criteria were used as indicators of having reached maximal exhaustion, such as reaching at least 95% of their individual predicted maximal HR calculated by the simple formula “220—Age (years)” (Fox et al., 1971) and reaching a respiratory quotient of >1.15 (Issekutz J et al., 1962). In the middle of each exercise stage, at 1.5 min, the RPE values on the scale 6 to 20 of each participant were obtained (Borg, 1970). MPO for each participant at each exercise stage was calculated with the formula: MPO (Watt) = BM (kg) × Gravitational Acceleration (9.81 m/s^2^) × Velocity (m/s) × Treadmill Grade (%). The workflow is visualized in Figure 1.

### Data analysis and statistics

#### Sample size calculation

Sample size was calculated from normative data on SMM (Janssen et al., 2000) and VO_2_max of healthy young adults (van der Steeg and Takken, 2021), indicating a desired number of at least n = 14 per group.

#### Anthropometric data, RHR, and test performance

Regarding anthropometric data (age, BM, height, BMI, FM, SMM) and RHR, normal distribution was tested with Shapiro-Wilk test (Shapiro and Wilk, 1965) and variance with Brown-Forsythe test for equal variance (Brown and Forsythe, 1974). The data were tested with unpaired two-tails Student’s t-tests (Student, 1908) for statistical difference for between group effects (Women *vs*. Men, and UT *vs*. T) and regarding data on exercise test performance: maximal performance time (min), VO_2_max (ml/kg/min), maximal MPO (Watt), maximal HR (bpm), and maximal RPE value. Effect sizes are stated as “Hedges’ g” values for differences between groups of unequal sample size (Hedges, 1981). For non-normally distributed data, non-parametric tests for unpaired data (Mann-Whitney Rank Sum test) were applied (Mann and Whitney, 1947).

#### Between group effects

Mean measured values of VO_2_, mean achieved MPO values, and mean RPE values were determined at each stage of the applied Bruce protocol. Mean achieved VO_2_ and MPO values were then calculated for each participant per BM and per SMM by dividing the respective VO_2_ and MPO values by the obtained values of BM and SMM of each participant. At each stage of the Bruce protocol (from rest to stage 7), these values were tested for differences across all four groups using Analysis of Variance (ANOVA) (Fisher, 1921) and *post hoc* Multiple Comparison Procedures (Holm-Šídák corrected tests) (Holm, 1979) to compare each group with every other group. Effect sizes are again stated as “Hedges’ g” values for differences between groups of unequal sample size, as well as the 95% confidence intervals of differences between the means. For non-normally distributed data, non-parametric tests (Kruskal-Wallis Rank Sum test) (Kruskal and Wallis, 1952) and non-parametric *post hoc* Multiple Comparison Procedures (Dunn’s corrected tests) were applied (Dunn, 1964).

#### Correlation analyses

Simple linear correlation analyses (Pearson) (Pearson, 1897) were performed with RPE values at each stage being the dependent variable and the mean values at each stage of VO_2_, VO_2_ per BM (VO_2BM_), VO_2_ per SMM (VO_2SMM_) and MPO, MPO per BM (MPO_BM_), MPO per SMM (MPO_SMM_), and HR, being the independent variables, respectively.

#### Bland-Altman analyses

All values of RPE, VO_2_, VO_2BM_, VO_2SMM_ and MPO, MPO_BM_, MPO_SMM_, and HR, obtained at each exercise stage, were standardized to calculate their “z-scores” by subtracting the mean of each parameter from all its raw values and subsequent division of the subtraction results by the standard deviation of each parameter, so that their mean was zero and their standard deviation was one (Mendenhall and Terry, 2007). This standardizing transformation allowed to subsequently perform the Bland-Altman method for agreement comparison between two methods of measurements (Bland and Altman, 1986), where the standardized values of RPE was method one and method two were the standardized values of VO_2_, VO_2BM_, VO_2SMM_ and MPO, MPO_BM_, MPO_SMM_, and HR, respectively. The absolute values of resulting differences between method one (RPE) and method two (VO_2_, VO_2BM_, VO_2SMM_ and MPO, MPO_BM_, MPO_SMM_) were then statistically compared with the absolute values of differences between RPE and HR through a *post hoc* Multiple Comparison Procedure (Holm-Šídák corrected tests).

For graphic representation, boxplots of MPO (absolute, per BM, and per SMM) and of RPE values were plotted to indicate group effects between the four groups (WU, WT, MU, MT) at each Bruce level. In addition, Bland-Altman plots were added for standardized values of RPE and MPO, RPE, and MPO_BM_, RPE and MPO_SMM_, and RPE and HR, to graphically represent their agreement. The statistical analyses and graphic representations were performed using “GraphPad Prism” for Windows, version 9.3.1 (GraphPad Software, San Diego, CA, United States); sample size and statistical power were calculated using “Systat SigmaPlot” for Windows, version 14.5 (Systat Software, San Jose, CA, United States). Data are reported as mean values ± standard deviation (SD) and statistical significance was assumed at a *p*-value of <0.05.

## Results

### Anthropometric data and resting heart rate

The anthropometric and RHR data are presented in Table 1. Compared to men, female participants exhibited a significantly lower BM, smaller height, greater percentage of FM and lower percentage of SMM. When comparing the untrained *versus* trained groups of both sexes, there were no differences for age, BM, height, and BMI. A difference was observed for RHR, with significantly lower values (>10 bpm) among the trained individuals, which corroborated the interview results taken to allocate the participants to either UT or T groups. Furthermore, percentages of FM and SMM were significantly different with lower values for FM and higher values for SMM for the trained groups.

**TABLE 1:** Anthropometric data and resting heart rate.

Parameter	Untrained (n = 34, w = 18, m = 16)	Trained (n = 29, w = 14, m = 15)	Hedges’ g (UT vs. T)	*p*-value (UT vs. T)
Age (years)				
All (mean ± SD)	26.18 ± 6.34	27.86 ± 5.19	0.288	0.157†
Women (mean ± SD)	24.94 ± 6.16	27.57 ± 5.02	0.462	0.123†
Men (mean ± SD)	27.56 ± 6.44	28.13 ± 5.51	0.095	0.793
Hedges’ g (Women *vs*. Men)	0.416	0.106		
*p*-value (Women *vs*. Men)	0.189†	0.777		
BM (kg)				
All (mean ± SD)	69.44 ± 11.87	72.44 ± 11.12	0.260	0.308
Women (mean ± SD)	62.00 ± 7.72	65.15 ± 8.95	0.381	0.294
Men (mean ± SD)	77.82 ± 10.06	79.25 ± 8.36	0.154	0.672
Hedges’ g (Women *vs*. Men)	1.779	1.630		
*p*-value (Women *vs*. Men)	**<0.001**	**<0.001**		
Height (cm)				
All (mean ± SD)	174.14 ± 9.99	175.22 ± 8.11	0.118	0.727
Women (mean ± SD)	166.94 ± 6.18	169.75 ± 6.86	0.443	0.234
Men (mean ± SD)	182.81 ± 5.83	180.33 ± 5.47	0.438	0.233
Hedges’ g (Women *vs*. Men)	2.637	1.712		
*p*-value (Women *vs*. Men)	**<0.001**	**<0.001**		
BMI (kg/m^2^)				
All (mean ± SD)	22.70 ± 2.48	23.46 ± 2.30	0.317	0.215
Women (mean ± SD)	22.21 ± 2.28	22.53 ± 2.27	0.141	0.698
Men (mean ± SD)	23.26 ± 2.64	24.33 ± 2.03	0.452	0.215
Hedges’ g (Women *vs*. Men)	0.428	0.838		
*p*-value (Women *vs*. Men)	0.224	**0.032**		
FM (%)				
All (mean ± SD)	21.91 ± 7.86	16.48 ± 5.66	0.783	**0.003**
Women (mean ± SD)	26.23 ± 5.93	19.69 ± 4.68	1.206	**0.001†**
Men (mean ± SD)	17.06 ± 6.96	13.49 ± 4.89	0.590	0.111
Hedges’ g (Women *vs*. Men)	1.425	1.294		
*p*-value (Women *vs*. Men)	**<0.001**	**0.002**		
SMM (%)				
All (mean ± SD)	43.38 ± 4.91	46.98 ± 3.71	0.818	**0.002**
Women (mean ± SD)	40.29 ± 3.31	44.47 ± 2.69	1.367	**<0.001†**
Men (mean ± SD)	46.85 ± 4.03	49.32 ± 2.93	0.697	0.062
Hedges’ g (Women *vs*. Men)	1.790	1.722		
*p*-value (Women *vs*. Men)	**<0.001**	**<0.001**		
RHR (bpm)				
All (mean ± SD)	74.97 ± 11.86	64.86 ± 11.15	0.876	**0.001**
Women (mean ± SD)	73.83 ± 11.65	63.57 ± 12.73	0.846	**0.024**
Men (mean ± SD)	76.25 ± 12.34	66.07 ± 9.74	0.912	**0.017†**
Hedges’ g (Women *vs*. Men)	0.202	0.222		
*p*-value (Women *vs*. Men)	0.561	0.557		

Anthropometric data and resting heart rate are stated as means ± SD. *p*-values and effect sizes through Hedges’ g are stated for differences between groups (UT *vs*. T) for all study participants and separately for women and men; and between sexes (Women *vs*. Men) for UT and T. † resembles results from non-parametric tests for non-normally distributed data. Statistically significant *p*-values printed in bold.

### Exercise test performance

All participants reached at least 95% of the predicted maximal heart rate, while a mean maximal respiratory quotient of 1.11 (±0.08) was reached. At absolute values, men achieved significantly greater maximal performance time, higher values of VO_2_max, and greater MPO than women. There were no differences between sexes for maximal attained RPE and maximal HR values among neither of the trained or untrained group, except for UT where men showed greater maximal HR than women. The trained group among both sexes achieved significantly greater maximal performance time, greater MPO, and higher values of VO_2_max than the untrained group. There were no significant differences between UT and T for maximal attained RPE and maximal HR values among neither sex. Exercise performance results are presented in Table 2. The number of participants to complete each exercise stage of the Bruce protocol before abandoning the test is shown in Supplementary Table S2.

**TABLE 2:** Exercise test performance.

Parameter	Untrained (n = 34, w = 18, m = 16)	Trained (n = 29, w = 14, m = 15)	Hedges’ g (UT vs. T)	*p*-value (UT vs. T)
Maximal Performance Time (min)				
All (mean ± SD)	15.85 ± 2.52	19.83 ± 2.23	1.664	**<0.001†**
Women (mean ± SD)	14.84 ± 1.65	18.39 ± 1.66	2.146	**<0.001**
Men (mean ± SD)	16.99 ± 2.88	21.18 ± 1.83	1.742	**<0.001†**
Hedges’ g (Women *vs*. Men)	0.931	1.594		
*p*-value (Women *vs*. Men)	**0.011†**	**<0.001†**		
VO_2_max (ml/kg/min)				
All (mean ± SD)	48.18 ± 7.02	57.03 ± 7.23	1.246	**<0.001†**
Women (mean ± SD)	44.44 ± 4.40	53.14 ± 5.02	1.859	**<0.001**
Men (mean ± SD)	52.38 ± 7.14	60.67 ± 7.20	1.156	**0.002†**
Hedges’ g (Women *vs*. Men)	1.358	1.206		
*p*-value (Women *vs*. Men)	**<0.001**	**0.002†**		
Maximal MPO (Watt)				
All (mean ± SD)	272.82 ± 68.38	380.18 ± 79.23	1.460	**<0.001**
Women (mean ± SD)	224.91 ± 40.41	311.51 ± 42.30	2.100	**<0.001**
Men (mean ± SD)	326.27 ± 50.81	444.27 ± 42.17	2.519	**<0.001**
Hedges’ g (Women *vs*. Men)	2.224	3.144		
*p*-value (Women *vs*. Men)	**<0.001**	**<0.001**		
Maximal Heart Rate (bpm)				
All (mean ± SD)	196.59 ± 8.69	192.00 ± 11.61	0.453	0.078
Women (mean ± SD)	193.61 ± 6.71	189.93 ± 14.00	0.350	0.333
Men (mean ± SD)	199.94 ± 9.62	193.94 ± 8.89	0.647	0.082
Hedges’ g (Women *vs*. Men)	0.772	0.345		
*p*-value (Women *vs*. Men)	**0.032**	0.363		
Maximal RPE Value				
All (mean ± SD)	18.50 ± 1.35	18.86 ± 1.16	0.284	0.306†
Women (mean ± SD)	18.11 ± 1.37	18.50 ± 1.45	0.278	0.444
Men (mean ± SD)	18.94 ± 1.24	19.20 ± 0.68	0.258	0.817†
Hedges’ g (Women *vs*. Men)	0.633	0.626		
*p*-value (Women *vs*. Men)	0.068†	0.196†		

Maximal performance time, maximal oxygen consumption, maximal mechanic power output, maximal heart rate, and maximal REP values achieved during the exercise test as means ± SD. *p*-values and effect sizes through Hedges’ g are stated for differences between groups (UT *vs*. T) for all study participants and separately for women and men; and between sexes (Women *vs*. Men) for UT and T. † resembles results from non-parametric tests for non-normally distributed data. Statistically significant *p*-values printed in bold.

### Between group effects

#### Oxygen consumption

The results of absolute VO_2_ indicate significantly greater values for men compared to women and for trained individuals compared to the untrained. Notably, calculated per BM, VO_2_ does not show significant differences between the groups except for stage 5. Calculated per SMM, the results of VO_2_ indicate significantly greater values for women compared to men and for untrained compared to the trained individuals, at test stages 1–4. Supplementary Table S3 shows the results of VO_2_ (absolute, per BM, and per SMM) for each group at each stage of the applied Bruce protocol and the results of the performed ANOVA for differences across all groups, while Supplementary Table S6–S8 show the results of the applied multi comparison analyses.

#### Mechanic power output

The results of absolute MPO indicate significantly greater values for men compared to women and for trained individuals compared to the untrained, at each stage of the exercise test. MPO_BM_ does not show significant differences between the groups, at none of the exercise stages.

**FIGURE 2:**
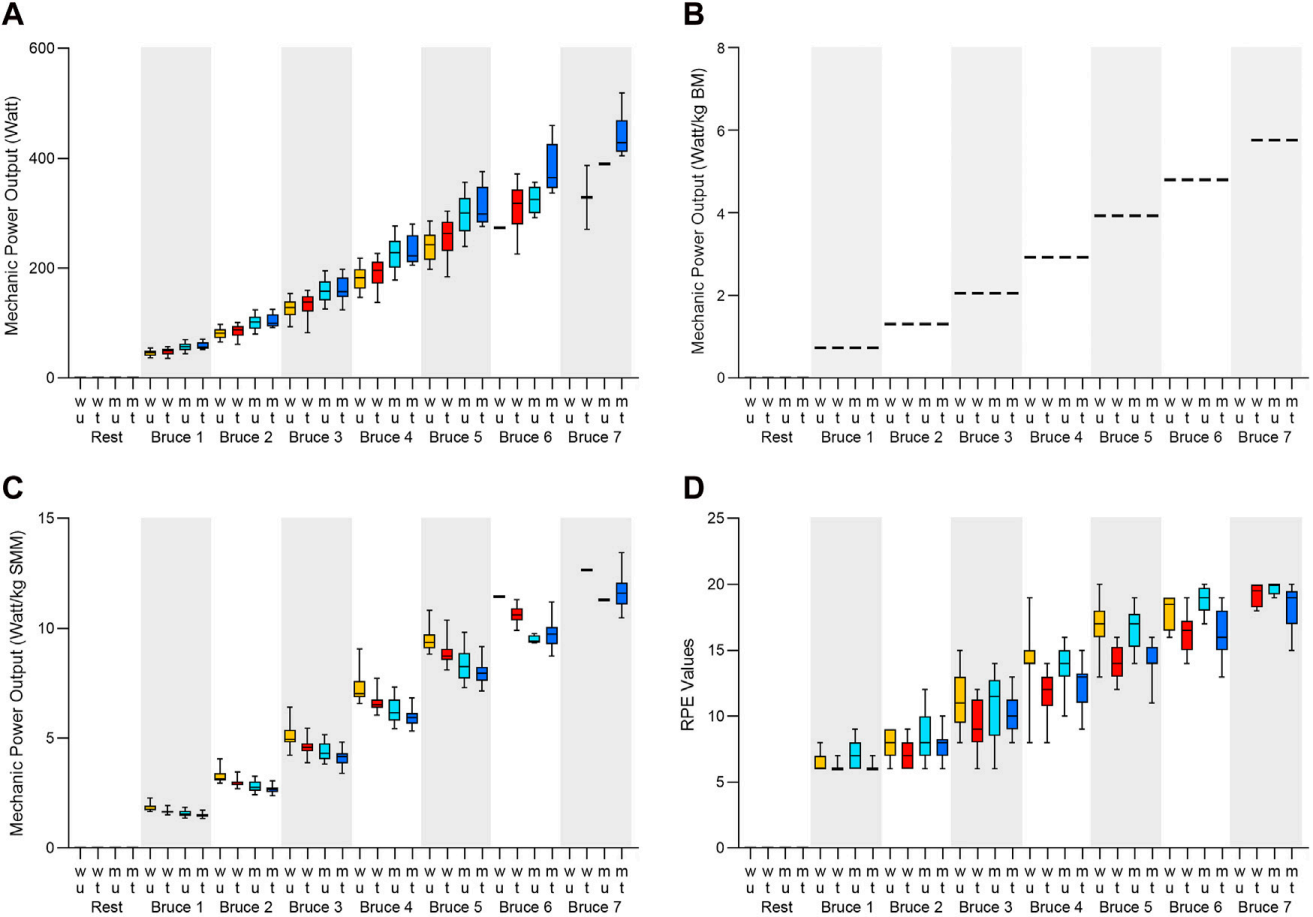
Boxplots of **(A)** MPO absolute, **(B)** MPO_BM_, **(C)** MPO_SMM_, and **(D)** of RPE Values, for between group effects at each stage of the Bruce protocol, with yellow boxes representing “Women Untrained” (WU), red boxes representing “Women Trained” (WT), cyan boxes representing “Men Untrained” (MU), and blue boxes representing “Men Trained” (MT). Boxes represent the 25th to 75th percentile range and median line within, while error bar whiskers represent the minimum and maximum ranges.

Results of MPO_SMM_ indicate significantly greater values for women compared to men and for untrained compared to the trained individuals, at test stages 1–6. Supplementary Table S4 shows the results of MPO (absolute, per BM, and per SMM) for each group at each stage of the Bruce protocol and the results of ANOVA for differences across all groups, while Supplementary Table S9–S11 show the results of multi comparison analyses (see Figure 2, panels A–C for a graphical representation of MPO results).

#### RPE values

The results of RPE indicate significantly greater values for the untrained compared to trained individuals, at the stages 1, 2, and 4 to 6. Supplementary Table S5 shows the results of obtained RPE for each group at each stage of the Bruce protocol and the results of ANOVA for differences across all groups, while Supplementary Table S12 shows the results of multi comparison analyses (see Figure 2, panel D for graphical representation of RPE results).

#### Correlation analyses

All *p*-values indicate a highly significant correlation between the RPE and the independent parameters. With regards to VO_2_ being the independent variable, the correlation coefficients (R) and coefficients of determination (R^2^) indicate a greater determination of the dependent variable “RPE” in dependence of “VO_2BM_” (R^2^ = 0.8103) when comparing it with the dependence of “VO_2_ absolute” (R^2^ = 0.7250) and a slightly greater value in dependence of “VO_2SMM_” (R^2^ = 0.8171). With regards to MPO being the independent variable, the coefficients of determination (R^2^) indicate a greater determination of the dependent variable “RPE” in dependence of “MPO_BM_” (R^2^ = 0.8176) when comparing it with the dependence of “MPO absolute” (R^2^ = 0.7609) and an even greater value in dependence of “MPO_SMM_” (R^2^ = 0.8345). The correlation between RPE and HR yielded an R^2^ of 0.7745. The results of the correlation analyses are shown in Table 3.

**TABLE 3:** Correlation between RPE values and VO_2_, mechanic power output, and heart rate.

Dependent parameter (Y)	Independent parameter (X)	Regression-equation	R	R^2^	*p*-value
RPE	VO_2_ absolute (l/min)	Y = 3.006*X + 4.441	0.8515	0.7250	**<0.0001**
RPE	VO_2BM_ (ml/kg BM/min)	Y = 0.2464*X + 3.405	0.9002	0.8103	**<0.0001**
RPE	VO_2SMM_ (ml/kg SMM/min)	Y = 0.1169*X + 3.089	0.9040	0.8171	**<0.0001**
RPE	MPO absolute (W)	Y = 0.03238*X + 5.538	0.8723	0.7609	**<0.0001**
RPE	MPO_BM_ (W/kg BM)	Y = 2.522*X + 4.997	0.9042	0.8176	**<0.0001**
RPE	MPO_SMM_ (W/kg SMM)	Y = 1.206*X + 4.697	0.9135	0.8345	**<0.0001**
RPE	HR (bpm)	Y = 0.1048*X—3.679	0.8801	0.7745	**<0.0001**

Results of the correlation analyses between the obtained RPE values (absolute) and VO_2_, VO_2_ per body mass, and VO_2_ per skeletal muscle mass, as well as mechanic power output, mechanic power output per body mass, and mechanic power output per skeletal muscle mass, and heart rate, respectively. The regression equation, the correlation coefficient (R), and the coefficient of determination (R^2^) are stated for each correlation. Statistically significant *p*-values printed in bold.

#### Bland-altman analyses

**FIGURE 3:**
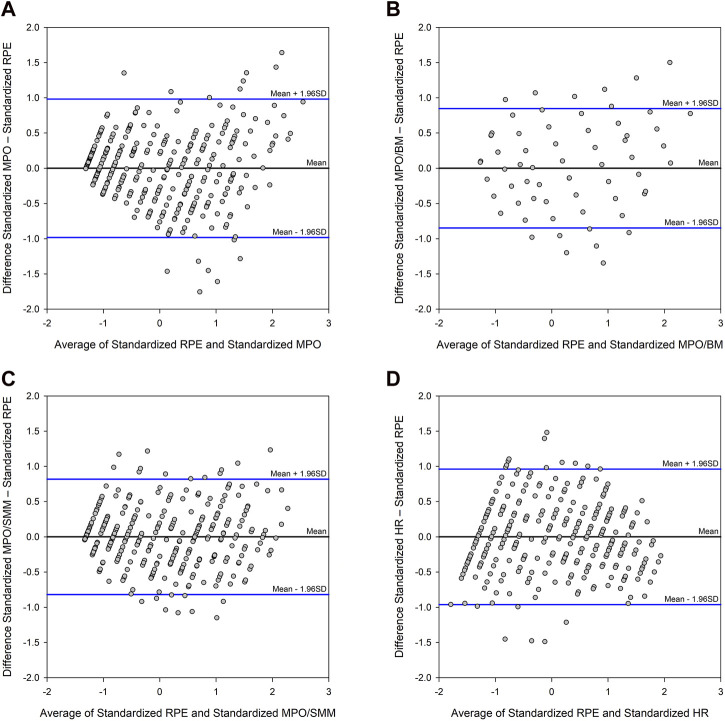
Bland-Altman plots between the standardized values of **(A)** PRE and MPO, **(B)** RPE and MPO_BM_, **(C)** RPE and MPO_SMM_, and **(D)** RPE and HR, by assigning the average of the two methods as the x-value, and the differences between the two methods as the y-value. The mean of the differences is displayed as a black line together with the limits of agreement for the difference data as blue lines at ±1.96 times the standard deviation of the differences.

The results of the Bland-Altman analyses of the standardized values are shown in Table 4 and reveal that the standard deviations of differences between RPE and VO_2_, and RPE, and MPO, respectively, are smallest when they are calculated per SMM. The standard deviations of differences between RPE and MPO_BM_ (0.4324) and MPO_SMM_ (0.4179) are significantly smaller than the standard deviation of differences between of RPE and HR (0.4905), which is graphically represented in Figure 3.

**TABLE 4:** Agreement between RPE values and VO_2_, mechanic power output, and heart rate.

Method one	Method two	SD of difference between method two—Method one	*p*-value of comparison with SD of difference standardized HR—Standardized RPE
Standardized RPE	Standardized VO_2_ (l/min)	0.5487	0.502
Standardized RPE	Standardized VO_2BM_ (ml/kg BM/min)	0.4478	0.095
Standardized RPE	Standardized VO_2SMM_ (ml/kg SMM/min)	0.4394	0.098
Standardized RPE	Standardized MPO (W)	0.5011	0.918
Standardized RPE	Standardized MPO_BM_ (W/kg BM)	0.4324	**0.039**
Standardized RPE	Standardized MPO_SMM_ (W/kg SMM)	0.4179	**0.008**
Standardized RPE	Standardized HR (bpm)	0.4905	—

Results of the Bland-Altman analyses as differences between the standardized RPE values (method one) and standardized values of VO_2_, VO_2_ per body mass, and VO_2_ per skeletal muscle mass, as well as standardized values of mechanic power output, mechanic power output per body mass, and mechanic power output per skeletal muscle mass, and heart rate, respectively (method two). The standard deviation of the differences between both methods is stated in column three. Statistically significant *p*-values printed in bold.

## Discussion

It was the aim of the study to evaluate the influence of sex and training status on individual RPE and its relation to objectively measurable physical strain parameters such as MPO and VO_2_ based on individual body composition at different exercise intensities to gain better insight on how healthy adults of different sex and training status perceive physical strain.

The three formulated hypotheses of this study were confirmed by the results 1) VO_2_ and MPO are higher in men compared to women and in trained compared to untrained participants, but when calculated per SMM, VO_2_ and MPO indicate opposite results already at low intensity stages; 2) RPE can be closely related to objectively measurable parameters of physical strain, like the MPO and VO_2_, and—as a key finding of this study—to a considerably greater degree when these parameters are calculated per SMM where RPE values had highest correlation with MPO per SMM compared to absolute MPO, or MPO per BM; and 3) within the 15-grade Borg scale, the agreement of RPE with MPO relative to SMM was higher than the agreement of RPE and HR.

Within each group, participants were of comparable age and RHR. There were no differences between the untrained and trained subjects regarding age, BM, height, and BMI, which can thusly be construed as comparable homogenous groups regarding these parameters. Among the trained, there were lower FM, higher SMM, and lower RHR, thus reflecting their better endurance training status (Kolnes et al., 2021; Pentikainen et al., 2021).

Having reached 95% of predicted maximal heart rates and a mean respiratory quotient of 1.11 along with the reported maximal RPE values was interpreted as having fulfilled the test to individual maximal exhaustion. Trained individuals showed longer maximal performance time, higher VO_2_max, and maximal MPO, which was to be expected from previous literature on test performance (Jones and Carter, 2000) and VO_2_max (Montero et al., 2015). When comparing sexes within each group, again, as expected, men showed longer maximal performance time, higher VO_2_max, and maximal MPO, compared to women (Besson et al., 2022).

The maximal reached HR did only differ significantly between men and women among the untrained group with greater values for men. This is, in line with previous findings that maximal HR does not change with endurance training or shows only small decreases (Zavorsky, 2000).

The maximal RPE values, i.e., the values of perceived exertion stated at peak performance, did neither differ between sexes nor trained and untrained groups. This observation is supported by previous findings for training status (Hill et al., 1987) or sex (Loe et al., 2013). These results come not as a surprise since each individual was at their individual peak performance. However, the differences in RPE between the untrained and trained groups as well as between sexes are considerable at submaximal performance, which has been shown before for RPE at intensities of lactate threshold (Demello et al., 1987), and which might be of interest for all athletes exercising at various intensity levels and especially trainers who wish to lay out individualized training recommendations.

The results show greater absolute values of VO_2_ and MPO for the trained and male participants at each Bruce level, with large to huge effect sizes (Cohen, 1998; Sawilowsky, 2009). Except for Bruce level 5, there were no differences between groups in VO_2BM_ at any given level; and—per definition—all values of MPO_BM_ were virtually the same for all participants at each Bruce level, because then only treadmill velocity and angle of gradient determine the load, which were the same for all participants at each Bruce level.

At submaximal performance, as indicated by Bruce level 1 to 4, values of VO_2SMM_ become greater for the female and untrained participants compared to the male and trained ones, with large to very large effect sizes (Cohen, 1998; Sawilowsky, 2009). This is also resembled—in impressive regularity shown in Figure 2C—by MPO_SMM_, at Bruce level 1 to 6, indicating a greater MPO_SMM_ for the female and untrained participants, again with large to huge effect sizes (Cohen, 1998; Sawilowsky, 2009). Consideration of the different body composition can explain this observation, since there is less SMM among WU of the study, leading to greater values of VO_2_ and MPO among them when calculated per SMM, indicating a greater strain per kilogram SMM at each exercise stage compared to the MT. These results are in contradiction with a previous study (Galan-Rioja et al., 2020), which however considered only experienced cyclists and triathletes and had found that body mass-based or lean body mass-based load can be used interchangeably in male compared to female athletes regardless of the applied exercise protocol. However, only experienced cyclists and triathletes were tested in that study. Some explanation for higher relative VO_2_ values among women can be found in previous works that have shown that women rely more on aerobic metabolism, especially fatty acid oxidation, at a given bout of exercise intensity than men (Tarnopolsky, 2000; Devries, 2016). This difference in preferred energy metabolism might enable women to perform—and endure—greater MPO per muscle mass through higher relative contribution of aerobic metabolism (Cheneviere et al., 2011), but also leads to greater ventilatory strain (Phillips et al., 2019). Another study found that values of body fat analysis was associated with race time among Ironman triathletes and ultra-cyclists (Rust et al., 2012), while another study with endurance athletes performing consecutive Deca Iron triathlons did not find an influence of body composition on race results (Knechtle et al., 2007). Yet another study had shown that higher performing female cyclists were lighter and leaner than their less successful peers (Haakonssen et al., 2016). However, these studies have not evaluated perceived exertion in conjunction with exercise performance and the influence of body composition. The results indicate that the impact of a given bout of exercise intensity is different between individuals depending on sex and the endurance training status.

The results show greater values of RPE per Bruce level for untrained participants compared to trained ones, with no differences between both sexes. Although studies regarding sex- and training status-based differences on perceived exertion are scarce, the results of this study are in line with previous works. Sex and training differences have been shown regarding the RPE at a given exercise intensity, where untrained women rated an exercise bout at 80% of their VO_2_max as subjectively more strenuous than trained women at their 80% VO_2_max and those rated it higher than trained men, respectively (Demello et al., 1987). In another study, healthy active women rated an exercise bout at 2 liters VO_2_ and at six MET (where MET resembles the energy expenditure at basic metabolic rate (Ainsworth et al., 1993)) as subjectively more strenuous than the male participants at the same intensities (Phillips et al., 2019). The latter has been attributed to greater ventilatory work and higher ratings of perceived dyspnea. Again, these studies have not evaluated perceived exertion in conjunction with exercise performance and the influence of body composition. Based on the results, it can be concluded that subjectively perceived exertion at a given bout of exercise intensity is dependent on body composition, with greater RPE values among untrained and female individuals.

This suggestion is supported by correlation analyses, revealing highly significant correlations with large effect sizes (Cohen, 1998) of the obtained RPE values with other independent parameters such as VO_2_ and MPO. Interestingly, the correlation coefficient and coefficient of determination (R^2^) are highest, when the correlation is calculated for VO_2SMM_ and MPO_SMM_, indicating muscle mass as the most relevant factor: while only 76.1% of the variation of the RPE values is explained by absolute MPO, 83.4% of the variation of obtained RPE values is explained by MPO_SMM_, while merely 77.5% of the variation of the RPE values is explained by HR, which is interesting since HR has been the basis for the RPE scale (Borg, 1970). These findings are corroborated by the Bland-Altman analyses.

Regarding the agreement between two methods, it has been noted that any two methods that measure the same parameter will show a good correlation (Bland and Altman, 1986; Giavarina, 2015), whereupon the Bland-Altman method was proposed to evaluate the agreement between such methods. The Bland-Altman method was employed, not to measure the congruency of two measurement approaches (RPE, MPO, and VO_2_ all measure different aspects and use different units of measurement) but as a mathematical tool to test the agreement of the obtained values in order to leverage and enhance the performed correlation analyses, since all correlation results were highly significant. After standardization of the obtained values and subsequent application of the Bland-Altman method, greatest agreement was shown between RPE and VO_2SMM_ and RPE and MPO_SMM_, respectively. Surprisingly, the agreements between RPE and MPO_BM_, and RPE and MPO_SMM_, respectively, were significantly greater than the one of RPE and HR, on which the RPE 15-grade scale was originally based upon (Borg, 1998). Therefore, it is suggested to include body composition assessment, in terms of SMM, to improve sensitivity and precision of RPE. As the results suggest, RPE appears to be closely related to physical strain of the skeletal muscle, leading to higher values of RPE at any given exercise stage among the female and untrained individuals compared to the trained and male ones.

These findings might have implications with regard to training recommendations (Milot et al., 2019), especially in the field of professional, recreational and health exercise (Garnacho-Castano et al., 2018), but also regarding occupational safety (Phillips et al., 2019). Finally, it indicates that individual body composition might have a considerable impact on perceived exertion. Individualized training based on endurance training status has been proposed before, such as through individualized training based on heart rates and heart rate reserves (Karvonen et al., 1957), or based on individual ventilatory thresholds. The results indicate that the inclusion of body composition analyses might facilitate the individualization of training prescriptions. In addition, through extrapolation of the submaximal relationship between perceived exertion and VO_2_ or exercise intensity to a theoretical endpoint, it is possible to estimate VO_2_max, time to exhaustion, and critical power (Faulkner et al., 2007; Nakamura et al., 2008; Lopes et al., 2016), which would be more precise if body composition is considered. Thus, it is proposed that VO_2_ and MPO are calculated per individual SMM to resemble individual strain more closely.

The results of this study might help to explain why untrained individuals might perceive a given bout of exercise as more strenuous (Hassmen, 1990), or why females would overestimate perceived exertion (Skatrud-Mickelson et al., 2011). This might not solely be based on mental states (Pessoa et al., 2022), where negative attitudes were associated with higher RPE (Basset et al., 2022), or based on the varying perception of external stimuli (Nethery, 2002). It also might not just be an association with quicker onset of lactate accumulation among the untrained (Demello et al., 1987), and the unfamiliarity towards a certain form of exercise (Hassmen, 1990). Instead, the results suggest that untrained individuals, especially untrained women, perform at a higher work rate per kilogram SMM at any given bout of exercise compared to better trained individuals, especially better trained men. Essentially, the data indicate that MPO_SMM_ more accurately represents the subjectively perceived strain at any given bout of exercise intensity than just the absolute MPO or MPO_BM_. The greater correlation and agreement between RPE and MPO_SMM_ underscores this connection between these two parameters. The presented study highlights the importance of skeletal muscle tissue during aerobic exercise, especially at submaximal intensities, besides the respiratory and cardiovascular system. Aerobic exercise-induced adaptions in skeletal muscle can be characterized, among many others (van der Zwaard et al., 2021), by enhanced fatigue resistance via modulation of substrate availability and the effects of metabolic end products (Hargreaves and Spriet, 2020), and by mitochondrial plasticity to accommodate alterations in energy demands (Philp et al., 2021). As the homeostatic status is progressively disrupted, it is plausible that the present results reflect a more efficient ability of the skeletal muscle to accommodate the gradually required oxygen flux during endurance performance, especially in large muscle fibers (van der Zwaard et al., 2021).

To summarize, the results regarding VO_2_, MPO, and RPE, indicate a greater strain for female and untrained individuals performing an all-out exercise test when calculated per BM and even better if calculated per SMM. MPO_SMM_ explains the obtained individual RPE values to a considerably greater degree than just absolute MPO or MPO_BM_. As the greatest agreement has been found between RPE and MPO_SMM_, it is suggested that RPE closely resembles mechanic muscular strain. Therefore, exercise recommendations and training guidelines should consider RPE, also at submaximal levels. It is recommended that untrained individuals (and especially untrained women) obtain a prior analysis of their body composition to be able to calculate MPO_SMM_ for generating individualized exercise guidelines to avoid over-exertion. On the other hand, the results underscore the validity of the RPE scale, and that this individual and subjective “inner voice” shows high correlations and agreements with other objective strain parameters, which warrants the use of the RPE during exercise testing, prescription, and performance.

## Limitations and outlook

All study participants were young and healthy adults, therefore the use on other populations of different age, or different health conditions might not be directly applicable, especially in patients encumbered by cardio-pulmonary limitations or obesity. Further studies seem warranted in other exercise settings as well as among populations other than young healthy adults. Further efforts should include body composition evaluations based on other means than bioimpedance analyses, which might be not readily available in each instance. In addition, future studies might be directed to investigate allometric exponents for concurrent size descriptors (e.g., height, body mass, SMM), such as proposed in a previous work (Nevill et al., 2004), along with other parameters such as sex and training status to explain inter-individuality in RPE, HR, VO_2_, and MPO.

## Data Availability

The raw data supporting the conclusion of this article will be made available by the authors, without undue reservation.

## Abbreviations

COPD, chronic obstructive pulmonary disease; ECG, electrocardiography; FM, fat mass; HR, heart rate; MPO, mechanic power output; MPO_BM_, mechanic power output per body mass; MPO_SMM_, Mechanic power output per skeletal muscle mass; MT, men, trained; MU, men, untrained; RHR, resting heart rate; RPE, rating of perceived exertion; VO_2_, oxygen consumption; VO_2BM_, oxygen consumption per body mass; VO_2_max, maximal oxygen consumption; VO_2SMM_, oxygen consumption per skeletal muscle mass; WT, women, trained; WU, women, untrained.
