# Supplementary material for: The relevance of body composition assessment for the rating of perceived exertion in trained and untrained women and men
Source: Front Physiol. 2023 Aug 1;14:1188802. doi: 10.3389/fphys.2023.1188802 (PMC10431604; doi:10.3389/fphys.2023.1188802)
Supplement: Supplementary file 1 [file Table1.docx]

**Supplementary Material**

**Supplementary Table S1 Bruce Protocol**

| Stage | Duration (min) | Speed (km/h) | Grade (%) | ≈ MET |
| --- | --- | --- | --- | --- |
| Rest | 3 | 0.0 | 0 | 2 |
| 1 | 6 | 2.7 | 10 | 5 |
| 2 | 9 | 4.0 | 12 | 7 |
| 3 | 12 | 5.4 | 14 | 10 |
| 4 | 15 | 6.7 | 16 | 13 |
| 5 | 18 | 8.0 | 18 | 16 |
| 6 | 21 | 8.8 | 20 | 18 |
| 7 | 24 | 9.6 | 22 | 20 |

Applied Bruce Protocol.^1^ Metabolic Equivalent (MET) equals the basal metabolic rate and oxygen consumption of 3.5 ml/ kg body mass/ min. Thus, increases in oxygen consumption and energy expenditure can be expressed as manifolds of “MET”.^2^

Two men (one trained and one untrained) achieved an added 8th stage of the Bruce protocol. The results were not included in the analyses between groups since these individual values did not allow further statistical group comparisons at that exercise stage; however, these values were included in the overall test performance, such as the maximal performance time and maximal oxygen consumption.

**SUPPLEMENTARY REFERENCES**

1. Bruce RA, Blackmon JR, Jones JW, Strait G. Exercising Testing in Adult Normal Subjects and Cardiac Patients. Pediatrics. 1963;32:SUPPL 742-756.

2. Ainsworth BE, Haskell WL, Leon AS, et al. Compendium of physical activities: classification of energy costs of human physical activities. Med Sci Sports Exerc. 1993;25(1):71-80.

**Supplementary Table S2 Number of Participants per Bruce Level**

|  | **Number of Participants** | | | |
| --- | --- | --- | --- | --- |
| **Bruce Level** | **Women Untrained (n=)** | **Women Trained (n=)** | **Men Untrained (n=)** | **Men Trained (n=)** |
| **Level 0 (Rest)** | 18 | 14 | 16 | 15 |
| **Level 1 (2.7 km/h, 10%)** | 18 | 14 | 16 | 15 |
| **Level 2 (4.0 km/h, 12%)** | 18 | 14 | 16 | 15 |
| **Level 3 (5.4 km/h, 14%)** | 17 | 14 | 16 | 15 |
| **Level 4 (6.7 km/h, 16%)** | 17 | 14 | 16 | 15 |
| **Level 5 (8.0 km/h, 18%)** | 12 | 14 | 15 | 15 |
| **Level 6 (8.8 km/h, 20%)** | 1 | 12 | 4 | 15 |
| **Level 7 (9.6 km/h, 22%)** | 0 | 2 | 2 | 12 |

Number of participants to complete each exercise stage of the Bruce protocol before abandoning the exercise test.

**Supplementary Table S3 VO_2_ (absolute, per BM, and per SMM)**

|  | **VO_2_ absolute (l/min)** | | | |  |
| --- | --- | --- | --- | --- | --- |
| **Bruce Level** | **Women Untrained** | **Women Trained** | **Men Untrained** | **Men Trained** | ANOVA p-value |
| **Level 0 (Rest)** | 0.323 ± 0.075 | 0.382 ± 0.063 | 0.415 ± 0.043 | 0.483 ± 0.086 | **<0.0001** |
| **Level 1 (2.7 km/h, 10%)** | 0.799 ± 0.131 | 0.889 ± 0.106 | 1.044 ± 0.160 | 1.045 ± 0.198 | **<0.0001** |
| **Level 2 (4.0 km/h, 12%)** | 1.108 ± 0.204 | 1.190 ± 0.169 | 1.433 ± 0.231 | 1.435 ± 0.186 | **<0.0001**† |
| **Level 3 (5.4 km/h, 14%)** | 1.751 ± 0.533 | 1.857 ± 0.272 | 2.171 ± 0.398 | 2.229 ± 0.257 | **0.0010** |
| **Level 4 (6.7 km/h, 16%)** | 2.247 ± 0.642 | 2.548 ± 0.309 | 3.169 ± 0.466 | 3.199 ± 0.415 | **<0.0001** |
| **Level 5 (8.0 km/h, 18%)** | 2.718 ± 0.369 | 3.037 ± 0.408 | 3.671 ± 0.448 | 3.895 ± 0.545 | **<0.0001** |
| **Level 6 (8.8 km/h, 20%)** | 2.907 | 3.289 ± 0.427 | 3.828 ± 0.671 | 4.352 ± 0.562 | **0.0001** |
| **Level 7 (9.6 km/h, 22%)** |  | 3.019 ± 0.679 | 3.929 ± 0.467 | 4.557 ± 0.553 | **0.0087** |
|  | **VO_2BM_ (ml/kg BM/min)** | | | |  |
| **Bruce Level** | **Women Untrained** | **Women Trained** | **Men Untrained** | **Men Trained** | ANOVA p-value |
| **Level 0 (Rest)** | 5.222 ± 1.083 | 6.071 ± 1.791 | 5.375 ± 0.599 | 6.133 ± 1.147 | 0.2078†‡ |
| **Level 1 (2.7 km/h, 10%)** | 12.889 ± 1.268 | 13.786 ± 1.567 | 13.438 ± 1.321 | 13.133 ± 1.499 | 0.3554‡ |
| **Level 2 (4.0 km/h, 12%)** | 17.833 ± 1.863 | 18.357 ± 1.797 | 18.375 ± 1.218 | 18.133 ± 1.668 | 0.4634†‡ |
| **Level 3 (5.4 km/h, 14%)** | 29.706 ± 2.986 | 28.643 ± 2.818 | 27.813 ± 2.674 | 28.333 ± 3.627 | 0.3706‡ |
| **Level 4 (6.7 km/h, 16%)** | 36.278 ± 9.079 | 39.286 ± 2.788 | 40.688 ± 2.493 | 40.400 ± 3.303 | 0.0932‡ |
| **Level 5 (8.0 km/h, 18%)** | 44.25 ± 2.419 | 46.714 ± 2.788 | 48.000 ± 3.669 | 49.067 ± 3.043 | **0.0020** |
| **Level 6 (8.8 km/h, 20%)** | 51.000 | 51.583 ± 4.172 | 56.25 ± 4.657 | 54.867 ± 3.074 | 0.0997‡ |
| **Level 7 (9.6 km/h, 22%)** |  | 53.000 ± 1.000 | 58.000 ± 5.000 | 59.083 ± 3.989 | 0.2209‡ |
|  | **VO_2SMM_ (ml/kg SMM/min)** | | | |  |
| **Bruce Level** | **Women Untrained** | **Women Trained** | **Men Untrained** | **Men Trained** | ANOVA p-value |
| **Level 0 (Rest)** | 12.897 ± 2.224 | 13.584 ± 3.714 | 11.505 ± 1.144 | 12.489 ± 2.525 | 0.1820† |
| **Level 1 (2.7 km/h, 10%)** | 32.120 ± 4.237 | 30.979 ± 3.348 | 28.913 ± 3.959 | 26.683 ± 3.154 | **0.0015**† |
| **Level 2 (4.0 km/h, 12%)** | 44.619 ± 6.527 | 41.319 ± 4.046 | 39.583 ± 4.926 | 36.842 ± 3.520 | **0.0013**† |
| **Level 3 (5.4 km/h, 14%)** | 74.747 ± 11.483 | 64.358 ± 5.773 | 60.025 ± 9.526 | 57.435 ± 6.203 | **<0.0001**† |
| **Level 4 (6.7 km/h, 16%)** | 94.641 ± 8.654 | 88.298 ± 5.642 | 87.354 ± 7.671 | 82.134 ± 7.603 | **0.0004** |
| **Level 5 (8.0 km/h, 18%)** | 106.570 ± 9.483 | 104.988 ± 5.333 | 102.016 ± 7.087 | 99.828 ± 8.725 | 0.1387‡ |
| **Level 6 (8.8 km/h, 20%)** | 121.632 | 113.999 ± 9.237 | 111.080 ± 7.540 | 111.623 ± 9.302 | 0.7072‡ |
| **Level 7 (9.6 km/h, 22%)** |  | 116.482 ± 2.116 | 113.906 ± 9.897 | 119.551 ± 11.460 | 0.7960‡ |

VO_2_ absolute, VO_2_ per body mass, and VO_2_ per skeletal muscle mass achieved by each group at each Bruce level as means ± SD. P-values are stated for differences across groups. † resembles results from non-parametric tests for non-normally distributed data. ‡ resembles a power below 80%. Statistically significant p-values printed in bold.

**Supplementary Table S4 Mechanic Power Output (absolute, per BM, and per SMM)**

|  | **MPO absolute (Watt)** | | | |  |
| --- | --- | --- | --- | --- | --- |
| **Bruce Level** | **Women Untrained** | **Women Trained** | **Men Untrained** | **Men Trained** | ANOVA p-value |
| **Level 0 (Rest)** | 0 ± 0 | 0 ± 0 | 0 ± 0 | 0 ± 0 | n/a |
| **Level 1 (2.7 km/h, 10%)** | 45.613 ± 5.676 | 48.004 ± 6.432 | 57.248 ± 7.400 | 58.303 ± 6.152 | **<0.0001** |
| **Level 2 (4.0 km/h, 12%)** | 81.093 ± 10.094 | 85.478 ± 11.961 | 101.785 ± 13.159 | 103.653 ± 10.938 | **<0.0001** |
| **Level 3 (5.4 km/h, 14%)** | 126.359 ± 17.347 | 133.344 ± 20.764 | 159.533 ± 20.641 | 160.428 ± 21.032 | **<0.0001** |
| **Level 4 (6.7 km/h, 16%)** | 181.112 ± 22.545 | 190.313 ± 26.149 | 227.322 ± 29.392 | 231.477 ± 24.425 | **<0.0001** |
| **Level 5 (8.0 km/h, 18%)** | 240.865 ± 29.077 | 255.648 ± 35.125 | 300.943 ± 36.549 | 310.961 ± 32.816 | **<0.0001** |
| **Level 6 (8.8 km/h, 20%)** | 273.37 | 307.502 ± 43.958 | 324.57 ± 26.244 | 380.066 ± 40.108 | **0.0003** |
| **Level 7 (9.6 km/h, 22%)** |  | 328.905 ± 82.611 | 389.915 ± 1.223 | 443.629 ± 40.904 | **0.0114** |
|  | **MPO_BM_ (Watt/kg BM)** | | | |  |
| **Bruce Level** | **Women Untrained** | **Women Trained** | **Men Untrained** | **Men Trained** | ANOVA p-value |
| **Level 0 (Rest)** | 0 ± 0 | 0 ± 0 | 0 ± 0 | 0 ± 0 | n/a |
| **Level 1 (2.7 km/h, 10%)** | 0.736 ± 0 | 0.736 ± 0 | 0.736 ± 0 | 0.736 ± 0 | 0.5349†‡ |
| **Level 2 (4.0 km/h, 12%)** | 1.308 ± 0 | 1.308 ± 0 | 1.308 ± 0 | 1.308 ± 0 | 0.3383‡ |
| **Level 3 (5.4 km/h, 14%)** | 2.060 ± 0 | 2.060 ± 0 | 2.060 ± 0 | 2.060 ± 0 | 0.2550†‡ |
| **Level 4 (6.7 km/h, 16%)** | 2.921 ± 0 | 2.921 ± 0 | 2.921 ± 0 | 2.921 ± 0 | 0.8103†‡ |
| **Level 5 (8.0 km/h, 18%)** | 3.924 ± 0 | 3.924 ± 0 | 3.924 ± 0 | 3.924 ± 0 | 0.4458‡ |
| **Level 6 (8.8 km/h, 20%)** | 4.796 | 4.796 ± 0 | 4.796 ± 0 | 4.796 ± 0 | 0.3015†‡ |
| **Level 7 (9.6 km/h, 22%)** |  | 5.755 ± 0 | 5.755 ± 0 | 5.755 ± 0 | >0.9999‡ |
|  | **MPO_SMM_ (Watt/kg SMM)** | | | |  |
| **Bruce Level** | **Women Untrained** | **Women Trained** | **Men Untrained** | **Men Trained** | ANOVA p-value |
| **Level 0 (Rest)** | 0 ± 0 | 0 ± 0 | 0 ± 0 | 0 ± 0 | n/a |
| **Level 1 (2.7 km/h, 10%)** | 1.839 ± 0.166 | 1.661 ± 0.103 | 1.582 ± 0.138 | 1.497 ± 0.091 | **<0.0001**† |
| **Level 2 (4.0 km/h, 12%)** | 3.269 ± 0.296 | 2.953 ± 0.193 | 2.812 ± 0.246 | 2.661 ± 0.161 | **<0.0001**† |
| **Level 3 (5.4 km/h, 14%)** | 5.110 ± 0.513 | 4.598 ± 0.351 | 4.409 ± 0.400 | 4.113 ± 0.349 | **<0.0001** |
| **Level 4 (6.7 km/h, 16%)** | 7.301 ± 0.660 | 6.580 ± 0.413 | 6.281 ± 0.550 | 5.942 ± 0.359 | **<0.0001**† |
| **Level 5 (8.0 km/h, 18%)** | 9.441 ± 0.539 | 8.839 ± 0.555 | 8.371 ± 0.714 | 7.982 ± 0.482 | **<0.0001**† |
| **Level 6 (8.8 km/h, 20%)** | 11.438 | 10.616 ± 0.424 | 9.483 ± 0.195 | 9.756 ± 0.589 | **<0.0001** |
| **Level 7 (9.6 km/h, 22%)** |  | 12.649 ± 0.013 | 11.302 ± 0.011 | 11.643 ± 0.775 | 0.1611‡ |

Mechanic power output absolute, Mechanic power output per body mass, and Mechanic power output per skeletal muscle mass achieved by each group at each Bruce level as means ± SD. P-values are stated for differences across groups. † resembles results from non-parametric tests for non-normally distributed data. ‡ resembles a power below 80%. Statistically significant p-values printed in bold.

**Supplementary Table S5 RPE Values**

|  | **RPE Values absolute** | | | |  |
| --- | --- | --- | --- | --- | --- |
| **Bruce Level** | **Women Untrained** | **Women Trained** | **Men Untrained** | **Men Trained** | ANOVA p-value |
| **Level 0 (Rest)** | 0 ± 0 | 0 ± 0 | 0 ± 0 | 0 ± 0 | n/a |
| **Level 1 (2.7 km/h, 10%)** | 6.412 ± 0.712 | 6.413 ± 0.363 | 6.938 ± 0.998 | 6.143 ± 0.363 | **0.0174**† |
| **Level 2 (4.0 km/h, 12%)** | 7.941 ± 1.029 | 7.154 ± 1.068 | 8.625 ± 1.857 | 7.714 ± 1.138 | **0.0364** |
| **Level 3 (5.4 km/h, 14%)** | 11.176 ± 1.944 | 9.429 ± 1.869 | 10.875 ± 0.446 | 10.071 ± 1.439 | 0.0745‡ |
| **Level 4 (6.7 km/h, 16%)** | 14.353 ± 2.473 | 11.714 ± 1.634 | 13.750 ± 1.693 | 12.357 ± 1.598 | **0.0004**† |
| **Level 5 (8.0 km/h, 18%)** | 17.133 ± 1.922 | 14.214 ± 1.369 | 16.5000 ± 1.461 | 14.357 ± 1.336 | **<0.0001** |
| **Level 6 (8.8 km/h, 20%)** | 18.000 | 16.357 ± 1.447 | 18.667 ± 1.073 | 16.357 ± 1.598 | **0.0002** |
| **Level 7 (9.6 km/h, 22%)** |  | 19.00 ± 0.414 | 19.50 ± 0.707 | 18.308 ± 1.750 | 0.5826†‡ |

RPE values achieved by each group at each Bruce level as means ± SD. P-values are stated for differences across groups. † resembles results from non-parametric tests for non-normally distributed data. ‡ resembles a power below 80%. Statistically significant p-values printed in bold.

**Supplementary Table S6 VO_2_ absolute (Multiple Comparison)**

| **Bruce Level** | **Women Untrained** | **Women Trained** | **Men Untrained** | **Men Trained** |  |  | |  | |  |  |  |
| --- | --- | --- | --- | --- | --- | --- | --- | --- | --- | --- | --- | --- |
| **Level 0 (Rest)** |  | | | | 95% Confidence Interval of Differences | | Hedges’ g | | Multiple Comparison p-value | |  |  |
|  | 0.323 ± 0.075 | 0.382 ± 0.063 |  |  | -0.1258 to 0.007959 | 0.842 | | 0.1030 | |  |  |  |
|  | 0.323 ± 0.075 |  | 0.415 ± 0.043 |  | -0.1558 to -0.02682 | 1.482 | | **0.0023** | |  |  |  |
|  | 0.323 ± 0.075 |  |  | 0.483 ± 0.086 | -0.2256 to -0.09439 | 1.996 | | **<0.0001** | |  |  |  |
|  |  | 0.382 ± 0.063 | 0.415 ± 0.043 |  | -0.1011 to 0.03629 | 0.620 | | 0.6001 | |  |  |  |
|  |  | 0.382 ± 0.063 |  | 0.483 ± 0.086 | -0.1708 to -0.03135 | 1.332 | | **0.0017** | |  |  |  |
|  |  |  | 0.415 ± 0.043 | 0.483 ± 0.086 | -0.1361 to -0.001265 | 1.011 | | **0.0443** | |  |  |  |
| **Level 1 (2.7 km/h, 10%)** |  | | | |  | |  | |  | |  |  |
|  | 0.799 ± 0.131 | 0.889 ± 0.106 |  |  | -0.2337 to 0.05349 | 0.745 | | 0.3544 | |  |  |  |
|  | 0.799 ± 0.131 |  | 1.044 ± 0.160 |  | -0.3826 to -0.1057 | 1.686 | | **0.0001** | |  |  |  |
|  | 0.799 ± 0.131 |  |  | 1.045 ± 0.198 | -0.3866 to -0.1049 | 1.494 | | **0.0001** | |  |  |  |
|  |  | 0.889 ± 0.106 | 1.044 ± 0.160 |  | -0.3015 to -0.006595 | 1.127 | | **0.0373** | |  |  |  |
|  |  | 0.889 ± 0.106 |  | 1.045 ± 0.198 | -0.3054 to -0.005898 | 0.972 | | **0.0387** | |  |  |  |
|  |  |  | 1.044 ± 0.160 | 1.045 ± 0.198 | -0.1464 to 0.1432 | 0.006 | | >0.9999 | |  |  |  |
| **Level 2 (4.0 km/h, 12%)** |  | | | |  | |  | |  | |  |  |
|  | 1.108 ± 0.204 | 1.190 ± 0.169 |  |  | -0.2708 to 0.1064 | 0.432 | | >0.9999† | |  |  |  |
|  | 1.108 ± 0.204 |  | 1.433 ± 0.231 |  | -0.5064 to -0.1427 | 1.492 | | **0.0012**† | |  |  |  |
|  | 1.108 ± 0.204 |  |  | 1.435 ± 0.186 | -0.5119 to -0.1419 | 1.668 | | **0.0004**† | |  |  |  |
|  |  | 1.190 ± 0.169 | 1.433 ± 0.231 |  | -0.4361 to -0.04872 | 1.194 | | 0.0676† | |  |  |  |
|  |  | 1.190 ± 0.169 |  | 1.435 ± 0.186 | -0.4414 to -0.04805 | 1.376 | | **0.0317**† | |  |  |  |
|  |  |  | 1.433 ± 0.231 | 1.435 ± 0.186 | -0.1925 to 0.1879 | 0.010 | | >0.9999† | |  |  |  |
| **Level 3 (5.4 km/h, 14%)** |  | | | |  | |  | |  | |  |  |
|  | 1.751 ± 0.533 | 1.857 ± 0.272 |  |  | -0.3067 to 0.2994 | 0.243 | | >0.9999 | |  |  |  |
|  | 1.751 ± 0.533 |  | 2.171 ± 0.398 |  | -0.6103 to -0.02538 | 0.889 | | **0.0281** | |  |  |  |
|  | 1.751 ± 0.533 |  |  | 2.229 ± 0.257 | -0.6737 to -0.07875 | 1.119 | | **0.0077** | |  |  |  |
|  |  | 1.857 ± 0.272 | 2.171 ± 0.398 |  | -0.6215 to -0.006926 | 0.909 | | **0.0432** | |  |  |  |
|  |  | 1.857 ± 0.272 |  | 2.229 ± 0.257 | -0.6846 to -0.06053 | 1.409 | | **0.0131** | |  |  |  |
|  |  |  | 2.171 ± 0.398 | 2.229 ± 0.257 | -0.3601 to 0.2434 | 0.172 | | 0.9561 | |  |  |  |
| **Level 4 (6.7 km/h, 16%)** |  | | | |  | |  | |  | |  |  |
|  | 2.247 ± 0.642 | 2.548 ± 0.309 |  |  | -0.5353 to 0.1992 | 0.579 | | 0.6227 | |  |  |  |
|  | 2.247 ± 0.642 |  | 3.169 ± 0.466 |  | -1.144 to -0.4349 | 1.635 | | **<0.0001** | |  |  |  |
|  | 2.247 ± 0.642 |  |  | 3.199 ± 0.415 | -1.180 to -0.4590 | 1.738 | | **<0.0001** | |  |  |  |
|  |  | 2.548 ± 0.309 | 3.169 ± 0.466 |  | -0.9936 to -0.2488 | 1.549 | | **0.0003** | |  |  |  |
|  |  | 2.548 ± 0.309 |  | 3.199 ± 0.415 | -1.030 to -0.2732 | 1.770 | | **0.0002** | |  |  |  |
|  |  |  | 3.169 ± 0.466 | 3.199 ± 0.415 | -0.3959 to 0.3355 | 0.068 | | 0.9963 | |  |  |  |
| **Level 5 (8.0 km/h, 18%)** |  | | | |  | |  | |  | |  |  |
|  | 2.718 ± 0.369 | 3.037 ± 0.408 |  |  | -0.7909 to 0.1533 | 0.817 | | 0.2885 | |  |  |  |
|  | 2.718 ± 0.369 |  | 3.671 ± 0.448 |  | -1.418 to -0.4890 | 2.296 | | **<0.0001** | |  |  |  |
|  | 2.718 ± 0.369 |  |  | 3.895 ± 0.545 | -1.642 to -0.7129 | 2.474 | | **<0.0001** | |  |  |  |
|  |  | 3.037 ± 0.408 | 3.671 ± 0.448 |  | -1.081 to -0.1890 | 1.477 | | **0.0022** | |  |  |  |
|  |  | 3.037 ± 0.408 |  | 3.895 ± 0.545 | -1.305 to -0.4129 | 1.773 | | **<0.0001** | |  |  |  |
|  |  |  | 3.671 ± 0.448 | 3.895 ± 0.545 | -0.6621 to 0.2143 | 0.449 | | 0.5321 | |  |  |  |
| **Level 6 (8.8 km/h, 20%)** |  | | | |  | |  | |  | |  |  |
|  | 2.907 | 3.289 ± 0.427 |  |  | -1.880 to 1.116 | 0.895 | | 0.8977 | |  |  |  |
|  | 2.907 |  | 3.828 ± 0.671 |  | -2.531 to 0.6879 | 1.373 | | 0.4151 | |  |  |  |
|  | 2.907 |  |  | 4.352 ± 0.562 | -2.932 to 0.04117 | 2.571 | | 0.0590 | |  |  |  |
|  |  | 3.289 ± 0.427 | 3.828 ± 0.671 |  | -1.370 to 0.2916 | 1.101 | | 0.3074 | |  |  |  |
|  |  | 3.289 ± 0.427 |  | 4.352 ± 0.562 | -1.621 to -0.5060 | 2.096 | | **<0.0001** | |  |  |  |
|  |  |  | 3.828 ± 0.671 | 4.352 ± 0.562 | -1.334 to 0.2860 | 0.899 | | 0.3102 | |  |  |  |
| **Level 7 (9.6 km/h, 22%)** |  | | | |  | |  | |  | |  |  |
|  |  |  |  |  |  |  | |  | |  |  |  |
|  |  |  |  |  |  |  | |  | |  |  |  |
|  |  |  |  |  |  |  | |  | |  |  |  |
|  |  | 3.019 ± 0.679 | 3.929 ± 0.467 |  | -2.383 to 0.5635 | 1.562 | | 0.2682 | |  |  |  |
|  |  | 3.019 ± 0.679 |  | 4.557 ± 0.553 | -2.664 to -0.4129 | 2.724 | | **0.0083** | |  |  |  |
|  |  |  | 3.929 ± 0.467 | 4.557 ± 0.553 | -1.754 to 0.4971 | 1.149 | | 0.3344 | |  |  |  |

VO_2_ absolute achieved by each group at each Bruce level as means ± SD. P-values, 95% confidence intervals of differences, and effect sizes through Hedges’ g are stated for differences between groups. † resembles results from non-parametric tests for non-normally distributed data. Statistically significant p-values printed in bold.

**Supplementary Table S7 VO_2BM_ (Multiple Comparison)**

| **Bruce Level** | **Women Untrained** | **Women Trained** | **Men Untrained** | **Men Trained** |  |  |  |  |  |
| --- | --- | --- | --- | --- | --- | --- | --- | --- | --- |
| **Level 0 (Rest)** |  | | | | 95% Confidence Interval of Differences | Hedges’ g | Multiple Comparison p-value |  |  |
|  | 5.222 ± 1.083 | 6.071 ± 1.791 |  |  | -2.023 to 0.3242 | 0.592 | >0.9999† |  |  |
|  | 5.222 ± 1.083 |  | 5.375 ± 0.599 |  | -1.284 to 0.9786 | 0.172 | >0.9999† |  |  |
|  | 5.222 ± 1.083 |  |  | 6.133 ± 1.147 | -2.062 to 0.2401 | 0.819 | 0.4123† |  |  |
|  |  | 6.071 ± 1.791 | 5.375 ± 0.599 |  | -0.5086 to 1.901 | 0.537 | >0.9999† |  |  |
|  |  | 6.071 ± 1.791 |  | 6.133 ± 1.147 | -1.286 to 1.162 | 0.042 | >0.9999† |  |  |
|  |  |  | 5.375 ± 0.599 | 6.133 ± 1.147 | -1.942 to 0.4251 | 0.837 | 0.3461† |  |  |
| **Level 1 (2.7 km/h, 10%)** |  | | | |  |  |  |  |  |
|  | 12.889 ± 1.268 | 13.786 ± 1.567 |  |  | -2.273 to 0.4789 | 0.638 | 0.3209 |  |  |
|  | 12.889 ± 1.268 |  | 13.438 ± 1.321 |  | -1.875 to 0.7779 | 0.425 | 0.6949 |  |  |
|  | 12.889 ± 1.268 |  |  | 13.133 ± 1.499 | -1.594 to 1.105 | 0.177 | 0.9635 |  |  |
|  |  | 13.786 ± 1.567 | 13.438 ± 1.321 |  | -1.065 to 1.761 | 0.242 | 0.9146 |  |  |
|  |  | 13.786 ± 1.567 |  | 13.133 ± 1.499 | -0.7823 to 2.087 | 0.426 | 0.6279 |  |  |
|  |  |  | 13.438 ± 1.321 | 13.133 ± 1.499 | -1.083 to 1.692 | 0.216 | 0.9378 |  |  |
| **Level 2 (4.0 km/h, 12%)** |  | | | |  |  |  |  |  |
|  | 17.833 ± 1.863 | 18.357 ± 1.797 |  |  | -2.138 to 1.090 | 0.286 | >0.9999† |  |  |
|  | 17.833 ± 1.863 |  | 18.375 ± 1.218 |  | -2.098 to 1.015 | 0.340 | 0.7117† |  |  |
|  | 17.833 ± 1.863 |  |  | 18.133 ± 1.668 | -1.884 to 1.284 | 0.169 | >0.9999† |  |  |
|  |  | 18.357 ± 1.797 | 18.375 ± 1.218 |  | -1.676 to 1.640 | 0.012 | >0.9999† |  |  |
|  |  | 18.357 ± 1.797 |  | 18.133 ± 1.668 | -1.459 to 1.907 | 0.129 | >0.9999† |  |  |
|  |  |  | 18.375 ± 1.218 | 18.133 ± 1.668 | -1.386 to 1.870 | 0.167 | >0.9999† |  |  |
| **Level 3 (5.4 km/h, 14%)** |  | | | |  |  |  |  |  |
|  | 29.706 ± 2.986 | 28.643 ± 2.818 |  |  | -1.941 to 4.068 | 0.365 | 0.7857 |  |  |
|  | 29.706 ± 2.986 |  | 27.813 ± 2.674 |  | -1.006 to 4.793 | 0.667 | 0.3192 |  |  |
|  | 29.706 ± 2.986 |  |  | 28.333 ± 3.627 | -1.577 to 4.322 | 0.416 | 0.6099 |  |  |
|  |  | 28.643 ± 2.818 | 27.813 ± 2.674 |  | -2.216 to 3.877 | 0.303 | 0.8884 |  |  |
|  |  | 28.643 ± 2.818 |  | 28.333 ± 3.627 | -2.784 to 3.403 | 0.095 | 0.9934 |  |  |
|  |  |  | 27.813 ± 2.674 | 28.333 ± 3.627 | -3.513 to 2.471 | 0.164 | 0.9673 |  |  |
| **Level 4 (6.7 km/h, 16%)** |  | | | |  |  |  |  |  |
|  | 36.278 ± 9.079 | 39.286 ± 2.788 |  |  | -3.568 to 1.820 | 0.430 | 0.8263 |  |  |
|  | 36.278 ± 9.079 |  | 40.688 ± 2.493 |  | -4.876 to 0.3246 | 0.653 | 0.1064 |  |  |
|  | 36.278 ± 9.079 |  |  | 40.4 ± 3.303 | -4.633 to 0.6564 | 0.589 | 0.2041 |  |  |
|  |  | 39.286 ± 2.788 | 40.688 ± 2.493 |  | -4.134 to 1.330 | 0.532 | 0.5310 |  |  |
|  |  | 39.286 ± 2.788 |  | 40.4 ± 3.303 | -3.889 to 1.660 | 0.363 | 0.7135 |  |  |
|  |  |  | 40.688 ± 2.493 | 40.4 ± 3.303 | -2.396 to 2.971 | 0.099 | 0.9920 |  |  |
| **Level 5 (8.0 km/h, 18%)** |  | | | |  |  |  |  |  |
|  | 44.25 ± 2.419 | 46.714 ± 2.788 |  |  | -5.766 to 0.8376 | 0.939 | 0.2084 |  |  |
|  | 44.25 ± 2.419 |  | 48 ± 3.669 |  | -7.001 to -0.4993 | 1.179 | **0.0177** |  |  |
|  | 44.25 ± 2.419 |  |  | 49.067 ± 3.043 | -8.067 to -1.566 | 1.729 | **0.0014** |  |  |
|  |  | 46.714 ± 2.788 | 48 ± 3.669 |  | -4.405 to 1.833 | 0.393 | 0.6947 |  |  |
|  |  | 46.714 ± 2.788 |  | 49.067 ± 3.043 | -5.471 to 0.7667 | 0.805 | 0.2006 |  |  |
|  |  |  | 48 ± 3.669 | 49.067 ± 3.043 | -4.131 to 1.998 | 0.317 | 0.7923 |  |  |
| **Level 6 (8.8 km/h, 20%)** |  | | | |  |  |  |  |  |
|  | 51.000 | 51.583 ± 4.172 |  |  | -11.82 to 10.65 | 0.140 | 0.9990 |  |  |
|  | 51.000 |  | 56.25 ± 4.657 |  | -17.32 to 6.815 | 1.127 | 0.6392 |  |  |
|  | 51.000 |  |  | 54.867 ± 3.074 | -15.01 to 7.279 | 1.258 | 0.7798 |  |  |
|  |  | 51.583 ± 4.172 | 56.25 ± 4.657 |  | -10.90 to 1.564 | 1.090 | 0.1961 |  |  |
|  |  | 51.583 ± 4.172 |  | 54.867 ± 3.074 | -7.463 to 0.8961 | 0.913 | 0.1639 |  |  |
|  |  |  | 56.25 ± 4.657 | 54.867 ± 3.074 | -4.689 to 7.456 | 0.406 | 0.9242 |  |  |
| **Level 7 (9.6 km/h, 22%)** |  | | | |  |  |  |  |  |
|  |  |  |  |  |  |  |  |  |  |
|  |  |  |  |  |  |  |  |  |  |
|  |  |  |  |  |  |  |  |  |  |
|  |  | 53.000 ± 1.000 | 58.000 ± 5.000 |  | -16.41 to 6.414 | 1.387 | 0.4982 |  |  |
|  |  | 53.000 ± 1.000 |  | 59.083 ± 3.989 | -14.80 to 2.634 | 1.588 | 0.1948 |  |  |
|  |  |  | 58.000 ± 5.000 | 59.083 ± 3.989 | -9.801 to 7.634 | 0.265 | 0.9426 |  |  |

VO_2_ per body mass achieved by each group at each Bruce level as means ± SD. P-values, 95% confidence intervals of differences, and effect sizes through Hedges’ g are stated for differences between groups. † resembles results from non-parametric tests for non-normally distributed data. Statistically significant p-values printed in bold.

**Supplementary Table S8 VO_2SMM_ (Multiple Comparison)**

| **Bruce Level** | **Women Untrained** | **Women Trained** | **Men Untrained** | **Men Trained** |  |  |  |  |  |
| --- | --- | --- | --- | --- | --- | --- | --- | --- | --- |
| **Level 0 (Rest)** |  | | | | 95% Confidence Interval of Differences | Hedges’ g | Multiple Comparison p-value |  |  |
|  | 12.897 ± 2.224 | 13.584 ± 3.714 |  |  | -3.136 to 1.762 | 0.232 | >0.9999† |  |  |
|  | 12.897 ± 2.224 |  | 11.505 ± 1.144 |  | -0.9688 to 3.753 | 0.773 | 0.2016† |  |  |
|  | 12.897 ± 2.224 |  |  | 12.489 ± 2.525 | -1.995 to 2.810 | 0.173 | >0.9999† |  |  |
|  |  | 13.584 ± 3.714 | 11.505 ± 1.144 |  | -0.4355 to 4.594 | 0.780 | 0.7650† |  |  |
|  |  | 13.584 ± 3.714 |  | 12.489 ± 2.525 | -1.459 to 3.648 | 0.347 | >0.9999† |  |  |
|  |  |  | 11.505 ± 1.144 | 12.489 ± 2.525 | -3.455 to 1.485 | 0.508 | >0.9999† |  |  |
| **Level 1 (2.7 km/h, 10%)** |  | | | |  |  |  |  |  |
|  | 32.120 ± 4.237 | 30.979 ± 3.348 |  |  | -2.408 to 4.869 | 0.294 | >0.9999† |  |  |
|  | 32.120 ± 4.237 |  | 28.913 ± 3.959 |  | -0.2100 to 6.807 | 0.780 | 0.2765† |  |  |
|  | 32.120 ± 4.237 |  |  | 26.683 ± 3.154 | 1.957 to 9.097 | 1.436 | **0.0016**† |  |  |
|  |  | 30.979 ± 3.348 | 28.913 ± 3.959 |  | -1.669 to 5.805 | 0.560 | >0.9999† |  |  |
|  |  | 30.979 ± 3.348 |  | 26.683 ± 3.154 | 0.5020 to 8.091 | 1.322 | **0.0199**† |  |  |
|  |  |  | 28.913 ± 3.959 | 26.683 ± 3.154 | -1.442 to 5.899 | 0.621 | 0.6060† |  |  |
| **Level 2 (4.0 km/h, 12%)** |  | | | |  |  |  |  |  |
|  | 44.619 ± 6.527 | 41.319 ± 4.046 |  |  | -1.626 to 8.226 | 0.590 | >0.9999† |  |  |
|  | 44.619 ± 6.527 |  | 39.583 ± 4.926 |  | 0.2862 to 9.785 | 0.864 | 0.1040† |  |  |
|  | 44.619 ± 6.527 |  |  | 36.842 ± 3.520 | 2.944 to 12.61 | 1.445 | **0.0007**† |  |  |
|  |  | 41.319 ± 4.046 | 39.583 ± 4.926 |  | -3.323 to 6.794 | 0.382 | >0.9999† |  |  |
|  |  | 41.319 ± 4.046 |  | 36.842 ± 3.520 | -0.6602 to 9.613 | 1.184 | 0.1132† |  |  |
|  |  |  | 39.583 ± 4.926 | 36.842 ± 3.520 | -2.227 to 7.709 | 0.637 | 0.8475† |  |  |
| **Level 3 (5.4 km/h, 14%)** |  | | | |  |  |  |  |  |
|  | 74.747 ± 11.483 | 64.358 ± 5.773 |  |  | 1.761 to 19.02 | 1.109 | 0.2465† |  |  |
|  | 74.747 ± 11.483 |  | 60.025 ± 9.526 |  | 6.395 to 23.05 | 1.391 | **0.0004**† |  |  |
|  | 74.747 ± 11.483 |  |  | 57.435 ± 6.203 | 8.843 to 25.78 | 1.843 | **<0.0001**† |  |  |
|  |  | 64.358 ± 5.773 | 60.025 ± 9.526 |  | -4.415 to 13.08 | 0.541 | 0.4332† |  |  |
|  |  | 64.358 ± 5.773 |  | 57.435 ± 6.203 | -1.960 to 15.81 | 1.154 | 0.1723† |  |  |
|  |  |  | 60.025 ± 9.526 | 57.435 ± 6.203 | -6.002 to 11.18 | 0.320 | >0.9999† |  |  |
| **Level 4 (6.7 km/h, 16%)** |  | | | |  |  |  |  |  |
|  | 94.641 ± 8.654 | 88.298 ± 5.642 |  |  | -1.102 to 13.79 | 0.851 | 0.1211 |  |  |
|  | 94.641 ± 8.654 |  | 87.354 ± 7.671 |  | 0.1009 to 14.47 | 0.889 | **0.0457** |  |  |
|  | 94.641 ± 8.654 |  |  | 82.134 ± 7.603 | 5.200 to 19.82 | 1.529 | **0.0002** |  |  |
|  |  | 88.298 ± 5.642 | 87.354 ± 7.671 |  | -6.607 to 8.492 | 0.139 | 0.9874 |  |  |
|  |  | 88.298 ± 5.642 |  | 82.134 ± 7.603 | -1.501 to 13.83 | 0.916 | 0.1567 |  |  |
|  |  |  | 87.354 ± 7.671 | 82.134 ± 7.603 | -2.192 to 12.64 | 0.683 | 0.2552 |  |  |
| **Level 5 (8.0 km/h, 18%)** |  | | | |  |  |  |  |  |
|  | 106.570 ± 9.483 | 104.988 ± 5.333 |  |  | -6.827 to 9.990 | 0.210 | 0.9589 |  |  |
|  | 106.570 ± 9.483 |  | 102.016 ± 7.087 |  | -3.725 to 12.83 | 0.553 | 0.4687 |  |  |
|  | 106.570 ± 9.483 |  |  | 99.828 ± 8.725 | -1.536 to 15.02 | 0.744 | 0.1477 |  |  |
|  |  | 104.988 ± 5.333 | 102.016 ± 7.087 |  | -4.971 to 10.91 | 0.471 | 0.7540 |  |  |
|  |  | 104.988 ± 5.333 |  | 99.828 ± 8.725 | -2.782 to 13.10 | 0.708 | 0.3217 |  |  |
|  |  |  | 102.016 ± 7.087 | 99.828 ± 8.725 | -5.616 to 9.993 | 0.275 | 0.8787 |  |  |
| **Level 6 (8.8 km/h, 20%)** |  | | | |  |  |  |  |  |
|  | 121.632 | 113.999 ± 9.237 |  |  | -19.49 to 34.75 | 0.826 | 0.8680 |  |  |
|  | 121.632 |  | 111.080 ± 7.540 |  | -18.58 to 39.68 | 1.399 | 0.7568 |  |  |
|  | 121.632 |  |  | 111.623 ± 9.302 | -16.90 to 36.91 | 1.076 | 0.7419 |  |  |
|  |  | 113.999 ± 9.237 | 111.080 ± 7.540 |  | -12.12 to 17.96 | 0.328 | 0.9511 |  |  |
|  |  | 113.999 ± 9.237 |  | 111.623 ± 9.302 | -7.715 to 12.47 | 0.256 | 0.9172 |  |  |
|  |  |  | 111.080 ± 7.540 | 111.623 ± 9.302 | -15.20 to 14.12 | 0.060 | 0.9996 |  |  |
| **Level 7 (9.6 km/h, 22%)** |  | | | |  |  |  |  |  |
|  |  |  |  |  |  |  |  |  |  |
|  |  |  |  |  |  |  |  |  |  |
|  |  |  |  |  |  |  |  |  |  |
|  |  | 116.482 ± 2.116 | 113.906 ± 9.897 |  | -28.32 to 33.48 | 0.360 | 0.9736 |  |  |
|  |  | 116.482 ± 2.116 |  | 119.551 ± 11.460 | -26.67 to 20.54 | 0.279 | 0.9375 |  |  |
|  |  |  | 113.906 ± 9.897 | 119.551 ± 11.460 | -29.25 to 17.96 | 0.492 | 0.8056 |  |  |

VO_2_ per skeletal muscle mass achieved by each group at each Bruce level as means ± SD. P-values, 95% confidence intervals of differences, and effect sizes through Hedges’ g are stated for differences between groups. † resembles results from non-parametric tests for non-normally distributed data. Statistically significant p-values printed in bold.

**Supplementary Table S9 Mechanic Power Output absolute (Multiple Comparison)**

| **Bruce Level** | **Women Untrained** | **Women Trained** | **Men Untrained** | **Men Trained** | |  |  |  |  |  |
| --- | --- | --- | --- | --- | --- | --- | --- | --- | --- | --- |
| **Level 0 (Rest)** |  | | | | 95% Confidence Interval of Differences | | Hedges’ g | Multiple Comparison p-value |  |  |
|  | 0 ± 0 | 0 ± 0 |  |  | | n/a | 0 | n/a |  |  |
|  | 0 ± 0 |  | 0 ± 0 |  | | n/a | 0 | n/a |  |  |
|  | 0 ± 0 |  |  | 0 ± 0 | | n/a | 0 | n/a |  |  |
|  |  | 0 ± 0 | 0 ± 0 |  | | n/a | 0 | n/a |  |  |
|  |  | 0 ± 0 |  | 0 ± 0 | | n/a | 0 | n/a |  |  |
|  |  |  | 0 ± 0 | 0 ± 0 | | n/a | 0 | n/a |  |  |
| **Level 1 (2.7 km/h, 10%)** |  | | | |  | |  |  |  |  |
|  | 45.613 ± 5.676 | 48.004 ± 6.432 |  |  | | -8.446 to 3.663 | 0.397 | 0.7242 |  |  |
|  | 45.613 ± 5.676 |  | 57.248 ± 7.400 |  | | -17.47 to -5.797 | 1.779 | **<0.0001** |  |  |
|  | 45.613 ± 5.676 |  |  | 58.303 ± 6.152 | | -18.63 to -6.751 | 2.152 | **<0.0001** |  |  |
|  |  | 48.004 ± 6.432 | 57.248 ± 7.400 |  | | -15.46 to -3.025 | 1.327 | **0.0013** |  |  |
|  |  | 48.004 ± 6.432 |  | 58.303 ± 6.152 | | -16.61 to -3.985 | 1.638 | **0.0004** |  |  |
|  |  |  | 57.248 ± 7.400 | 58.303 ± 6.152 | | -7.162 to 5.051 | 0.155 | 0.9680 |  |  |
| **Level 2 (4.0 km/h, 12%)** |  | | | |  | |  |  |  |  |
|  | 81.093 ± 10.094 | 85.478 ± 11.961 |  |  | | -15.21 to 6.543 | 0.489 | 0.7188 |  |  |
|  | 81.093 ± 10.094 |  | 101.785 ± 13.159 |  | | -31.18 to -10.20 | 2.087 | **<0.0001** |  |  |
|  | 81.093 ± 10.094 |  |  | 113.653 ± 10.938 | | -33.23 to -11.89 | 3.845 | **<0.0001** |  |  |
|  |  | 85.478 ± 11.961 | 101.785 ± 13.159 |  | | -27.53 to -5.186 | 1.292 | **0.0015** |  |  |
|  |  | 85.478 ± 11.961 |  | 113.653 ± 10.938 | | -29.57 to -6.882 | 2.462 | **0.0004** |  |  |
|  |  |  | 101.785 ± 13.159 | 113.653 ± 10.938 | | -12.84 to 9.102 | 0.978 | 0.9693 |  |  |
| **Level 3 (5.4 km/h, 14%)** |  | | | |  | |  |  |  |  |
|  | 126.359 ± 17.347 | 133.344 ± 20.764 |  |  | | -25.21 to 12.24 | 0.369 | 0.7965 |  |  |
|  | 126.359 ± 17.347 |  | 159.533 ± 20.641 |  | | -50.73 to -14.62 | 1.745 | **<0.0001** |  |  |
|  | 126.359 ± 17.347 |  |  | 160.428 ± 21.032 | | -51.94 to -15.20 | 1.779 | **<0.0001** |  |  |
|  |  | 133.344 ± 20.764 | 159.533 ± 20.641 |  | | -45.42 to -6.960 | 1.265 | **0.0036** |  |  |
|  |  | 133.344 ± 20.764 |  | 160.428 ± 21.032 | | -46.61 to -7.558 | 1.296 | **0.0029** |  |  |
|  |  |  | 159.533 ± 20.641 | 160.428 ± 21.032 | | -19.78 to 17.99 | 0.043 | 0.9993 |  |  |
| **Level 4 (6.7 km/h, 16%)** |  | | | |  | |  |  |  |  |
|  | 181.112 ± 22.545 | 190.313 ± 26.149 |  |  | | -33.37 to 14.97 | 0.380 | 0.7463 |  |  |
|  | 181.112 ± 22.545 |  | 227.322 ± 29.392 |  | | -69.52 to -22.90 | 1.772 | **<0.0001** |  |  |
|  | 181.112 ± 22.545 |  |  | 231.477 ± 24.425 | | -74.08 to -26.65 | 2.149 | **<0.0001** |  |  |
|  |  | 190.313 ± 26.149 | 227.322 ± 29.392 |  | | -61.83 to -12.18 | 1.329 | **0.0012** |  |  |
|  |  | 190.313 ± 26.149 |  | 231.477 ± 24.425 | | -66.37 to -15.96 | 1.629 | **0.0003** |  |  |
|  |  |  | 227.322 ± 29.392 | 231.477 ± 24.425 | | -28.53 to 20.22 | 0.153 | 0.9692 |  |  |
| **Level 5 (8.0 km/h, 18%)** |  | | | |  | |  |  |  |  |
|  | 240.865 ± 29.077 | 255.648 ± 35.125 |  |  | | -50.63 to 20.00 | 0.455 | 0.6600 |  |  |
|  | 240.865 ± 29.077 |  | 300.943 ± 36.549 |  | | -94.85 to -25.31 | 1.795 | **0.0002** |  |  |
|  | 240.865 ± 29.077 |  |  | 310.961 ± 32.816 | | -104.9 to -35.33 | 2.245 | **<0.0001** |  |  |
|  |  | 255.648 ± 35.125 | 300.943 ± 36.549 |  | | -78.12 to -11.40 | 1.263 | **0.0043** |  |  |
|  |  | 255.648 ± 35.125 |  | 310.961 ± 32.816 | | -88.14 to -21.42 | 1.629 | **0.0004** |  |  |
|  |  |  | 300.943 ± 36.549 | 310.961 ± 32.816 | | -42.80 to 22.76 | 0.288 | 0.8489 |  |  |
| **Level 6 (8.8 km/h, 20%)** |  | | | |  | |  |  |  |  |
|  | 273.370 | 307.502 ± 43.958 |  |  | | -149.1 to 80.85 | 0.776 | 0.8490 |  |  |
|  | 273.370 |  | 324.57 ± 26.244 |  | | -174.7 to 72.32 | 1.951 | 0.6734 |  |  |
|  | 273.370 |  |  | 380.066 ± 40.108 | | -220.8 to 7.403 | 2.660 | 0.0732 |  |  |
|  |  | 307.502 ± 43.958 | 324.57 ± 26.244 |  | | -80.85 to 46.72 | 0.418 | 0.8840 |  |  |
|  |  | 307.502 ± 43.958 |  | 380.066 ± 40.108 | | -115.4 to -29.78 | 1.734 | **0.0004** |  |  |
|  |  |  | 324.57 ± 26.244 | 380.066 ± 40.108 | | -117.7 to 6.672 | 1.459 | 0.0930 |  |  |
| **Level 7 (9.6 km/h, 22%)** |  | | | |  | |  |  |  |  |
|  |  |  |  |  | |  |  |  |  |  |
|  |  |  |  |  | |  |  |  |  |  |
|  |  |  |  |  | |  |  |  |  |  |
|  |  | 328.905 ± 82.611 | 389.915 ± 1.223 |  | | -177.3 to 55.31 | 1.044 | 0.3768 |  |  |
|  |  | 328.905 ± 82.611 |  | 443.629 ± 40.904 | | -203.6 to -25.88 | 2.502 | **0.0121** |  |  |
|  |  |  | 389.915 ± 1.223 | 443.629 ± 40.904 | | -142.6 to 35.13 | 1.327 | 0.2819 |  |  |

Mechanic power output absolute achieved by each group at each Bruce level as means ± SD. P-values, 95% confidence intervals of differences, and effect sizes through Hedges’ g are stated for differences between groups. Statistically significant p-values printed in bold.

**Supplementary Table S10 Mechanic Power Output per BM (Multiple Comparison)**

| **Bruce Level** | **Women Untrained** | **Women Trained** | **Men Untrained** | **Men Trained** |  |  |  |
| --- | --- | --- | --- | --- | --- | --- | --- |
| **Level 0 (Rest)** |  | | | | 95% Confidence  Interval of Differences | Hedges’ g | Multiple Comparison p-value |
|  | 0 ± 0 | 0 ± 0 |  |  | n/a | 0 | n/a |
|  | 0 ± 0 |  | 0 ± 0 |  | n/a | 0 | n/a |
|  | 0 ± 0 |  |  | 0 ± 0 | n/a | 0 | n/a |
|  |  | 0 ± 0 | 0 ± 0 |  | n/a | 0 | n/a |
|  |  | 0 ± 0 |  | 0 ± 0 | n/a | 0 | n/a |
|  |  |  | 0 ± 0 | 0 ± 0 | n/a | 0 | n/a |
| **Level 1 (2.7 km/h, 10%)** |  | | | |  |  |  |
|  | 0.736 ± 0 | 0.736 ± 0 |  |  | -0.0002094 to 0.0001104 | 0 | >0.9999† |
|  | 0.736 ± 0 |  | 0.736 ± 0 |  | -0.0001156 to 0.0001927 | 0 | >0.9999† |
|  | 0.736 ± 0 |  |  | 0.736 ± 0 | -0.0001831 to 0.0001306 | 0 | >0.9999† |
|  |  | 0.736 ± 0 | 0.736 ± 0 |  | -7.610e-005 to 0.0002523 | 0 | >0.9999† |
|  |  | 0.736 ± 0 |  | 0.736 ± 0 | -0.0001435 to 0.0001900 | 0 | >0.9999† |
|  |  |  | 0.736 ± 0 | 0.736 ± 0 | -0.0002261 to 9.640e-005 | 0 | >0.9999† |
| **Level 2 (4.0 km/h, 12%)** |  | | | |  |  |  |
|  | 1.308 ± 0 | 1.308 ± 0 |  |  | -7.417e-005 to 1.678e-005 | 0 | 0.3496 |
|  | 1.308 ± 0 |  | 1.308 ± 0 |  | -6.092e-005 to 2.678e-005 | 0 | 0.7333 |
|  | 1.308 ± 0 |  |  | 1.308 ± 0 | -6.937e-005 to 1.987e-005 | 0 | 0.4639 |
|  |  | 1.308 ± 0 | 1.308 ± 0 |  | -3.508e-005 to 5.833e-005 | 0 | 0.9122 |
|  |  | 1.308 ± 0 |  | 1.308 ± 0 | -4.348e-005 to 5.137e-005 | 0 | 0.9962 |
|  |  |  | 1.308 ± 0 | 1.308 ± 0 | -5.355e-005 to 3.818e-005 | 0 | 0.9707 |
| **Level 3 (5.4 km/h, 14%)** |  | | | |  |  |  |
|  | 2.060 ± 0 | 2.060 ± 0 |  |  | -6.908e-009 to 4.659e-008 | 0 | 0.5615† |
|  | 2.060 ± 0 |  | 2.060 ± 0 |  | -1.836e-008 to 3.322e-008 | 0 | >0.9999† |
|  | 2.060 ± 0 |  |  | 2.060 ± 0 | -7.354e-009 to 4.513e-008 | 0 | >0.9999† |
|  |  | 2.060 ± 0 | 2.060 ± 0 |  | -3.988e-008 to 1.506e-008 | 0 | 0.4654† |
|  |  | 2.060 ± 0 |  | 2.060 ± 0 | -2.885e-008 to 2.694e-008 | 0 | 0.7126† |
|  |  |  | 2.060 ± 0 | 2.060 ± 0 | -1.552e-008 to 3.844e-008 | 0 | >0.9999† |
| **Level 4 (6.7 km/h, 16%)** |  | | | |  |  |  |
|  | 2.921 ± 0 | 2.921 ± 0 |  |  | -0.0003354 to 0.0003565 | 0 | >0.9999† |
|  | 2.921 ± 0 |  | 2.921 ± 0 |  | -0.0003455 to 0.0003216 | 0 | >0.9999† |
|  | 2.921 ± 0 |  |  | 2.921 ± 0 | -0.0001589 to 0.0005199 | 0 | >0.9999† |
|  |  | 2.921 ± 0 | 2.921 ± 0 |  | -0.0003777 to 0.0003328 | 0 | >0.9999† |
|  |  | 2.921 ± 0 |  | 2.921 ± 0 | -0.0001908 to 0.0005307 | 0 | >0.9999† |
|  |  |  | 2.921 ± 0 | 2.921 ± 0 | -0.0001565 to 0.0005414 | 0 | >0.9999† |
| **Level 5 (8.0 km/h, 18%)** |  | | | |  |  |  |
|  | 3.924 ± 0 | 3.924 ± 0 |  |  | -0.0001009 to 2.466e-005 | 0 | >0.9999† |
|  | 3.924 ± 0 |  | 3.924 ± 0 |  | -8.377e-005 to 3.989e-005 | 0 | >0.9999† |
|  | 3.924 ± 0 |  |  | 3.924 ± 0 | -7.695e-005 to 4.671e-005 | 0 | >0.9999† |
|  |  | 3.924 ± 0 | 3.924 ± 0 |  | -4.312e-005 to 7.553e-005 | 0 | 0.3822† |
|  |  | 3.924 ± 0 |  | 3.924 ± 0 | -3.630e-005 to 8.235e-005 | 0 | >0.9999† |
|  |  |  | 3.924 ± 0 | 3.924 ± 0 | -5.147e-005 to 6.511e-005 | 0 | >0.9999† |
| **Level 6 (8.8 km/h, 20%)** |  | | | |  |  |  |
|  | 4.796 | 4.796 ± 0 |  |  | -0.0001185 to 0.0001068 | 0 | 0.9990 |
|  | 4.796 |  | 4.796 ± 0 |  | -0.0001688 to 7.315e-005 | 0 | 0.7048 |
|  | 4.796 |  |  | 4.796 ± 0 | -0.0001334 to 9.006e-005 | 0 | 0.9512 |
|  |  | 4.796 ± 0 | 4.796 ± 0 |  | -0.0001044 to 2.049e-005 | 0 | 0.2789 |
|  |  | 4.796 ± 0 |  | 4.796 ± 0 | -5.773e-005 to 2.607e-005 | 0 | 0.7327 |
|  |  |  | 4.796 ± 0 | 4.796 ± 0 | -3.474e-005 to 8.702e-005 | 0 | 0.6488 |
| **Level 7 (9.6 km/h, 22%)** |  | | | |  |  |  |
|  |  |  |  |  |  |  |  |
|  |  |  |  |  |  |  |  |
|  |  |  |  |  |  |  |  |
|  |  | 5.755 ± 0 | 5.755 ± 0 |  | -0.0001989 to 2.581e-005 | 0 | 0.9183 |
|  |  | 5.755 ± 0 |  | 5.755 ± 0 | -0.0001596 to 1.209e-005 | 0 | 0.9183 |
|  |  |  | 5.755 ± 0 | 5.755 ± 0 | -7.300e-005 to 9.865e-005 | 0 | 0.9183 |

Mechanic power output per body mass achieved by each group at each Bruce level as means ± SD. P-values, 95% confidence intervals of differences, and effect sizes through Hedges’ g are stated for differences between groups. † resembles results from non-parametric tests for non-normally distributed data. Statistically significant p-values printed in bold.

**Supplementary Table S11 Mechanic Power Output per SMM (Multiple Comparison)**

| **Bruce Level** | **Women Untrained** | **Women Trained** | **Men Untrained** | **Men Trained** |  |  |  |  |  |
| --- | --- | --- | --- | --- | --- | --- | --- | --- | --- |
| **Level 0 (Rest)** |  | | | | 95% Confidence Interval of Differences | Hedges’ g | Multiple Comparison p-value |  |  |
|  | 0 ± 0 | 0 ± 0 |  |  | n/a | 0 | n/a |  |  |
|  | 0 ± 0 |  | 0 ± 0 |  | n/a | 0 | n/a |  |  |
|  | 0 ± 0 |  |  | 0 ± 0 | n/a | 0 | n/a |  |  |
|  |  | 0 ± 0 | 0 ± 0 |  | n/a | 0 | n/a |  |  |
|  |  | 0 ± 0 |  | 0 ± 0 | n/a | 0 | n/a |  |  |
|  |  |  | 0 ± 0 | 0 ± 0 | n/a | 0 | n/a |  |  |
| **Level 1 (2.7 km/h, 10%)** |  | | | |  |  |  |  |  |
|  | 1.839 ± 0.166 | 1.661 ± 0.103 |  |  | 0.05486 to 0.3014 | 1.252 | 0.0579† |  |  |
|  | 1.839 ± 0.166 |  | 1.582 ± 0.138 |  | 0.1381 to 0.3758 | 1.674 | **0.0003**† |  |  |
|  | 1.839 ± 0.166 |  |  | 1.497 ± 0.091 | 0.2212 to 0.4631 | 2.491 | **<0.0001**† |  |  |
|  |  | 1.661 ± 0.103 | 1.582 ± 0.138 |  | -0.04776 to 0.2054 | 0.642 | >0.9999† |  |  |
|  |  | 1.661 ± 0.103 |  | 1.497 ± 0.091 | 0.03545 to 0.2926 | 1.691 | **0.0290**† |  |  |
|  |  |  | 1.582 ± 0.138 | 1.497 ± 0.091 | -0.03916 to 0.2095 | 0.722 | 0.6498† |  |  |
| **Level 2 (4.0 km/h, 12%)** |  | | | |  |  |  |  |  |
|  | 3.269 ± 0.296 | 2.953 ± 0.193 |  |  | 0.09509 to 0.5366 | 1.232 | 0.0742† |  |  |
|  | 3.269 ± 0.296 |  | 2.812 ± 0.246 |  | 0.2438 to 0.6695 | 1.670 | **0.0004**† |  |  |
|  | 3.269 ± 0.296 |  |  | 2.661 ± 0.161 | 0.3917 to 0.8249 | 2.487 | **<0.0001**† |  |  |
|  |  | 2.953 ± 0.193 | 2.812 ± 0.246 |  | -0.08596 to 0.3675 | 0.632 | >0.9999† |  |  |
|  |  | 2.953 ± 0.193 |  | 2.661 ± 0.161 | 0.06221 to 0.5227 | 1.649 | **0.0254**† |  |  |
|  |  |  | 2.812 ± 0.246 | 2.661 ± 0.161 | -0.07099 to 0.3743 | 0.721 | 0.6374† |  |  |
| **Level 3 (5.4 km/h, 14%)** |  | | | |  |  |  |  |  |
|  | 5.110 ± 0.513 | 4.598 ± 0.351 |  |  | 0.1199 to 0.9028 | 1.144 | **0.0055** |  |  |
|  | 5.110 ± 0.513 |  | 4.409 ± 0.400 |  | 0.3228 to 1.078 | 1.518 | **<0.0001** |  |  |
|  | 5.110 ± 0.513 |  |  | 4.113 ± 0.349 | 0.6129 to 1.381 | 2.245 | **<0.0001** |  |  |
|  |  | 4.598 ± 0.351 | 4.409 ± 0.400 |  | -0.2131 to 0.5909 | 0.500 | 0.6029 |  |  |
|  |  | 4.598 ± 0.351 |  | 4.113 ± 0.349 | 0.07743 to 0.8939 | 1.386 | **0.0135** |  |  |
|  |  |  | 4.409 ± 0.400 | 4.113 ± 0.349 | -0.09805 to 0.6916 | 0.787 | 0.2044 |  |  |
| **Level 4 (6.7 km/h, 16%)** |  | | | |  |  |  |  |  |
|  | 7.301 ± 0.660 | 6.580 ± 0.413 |  |  | 0.2308 to 1.211 | 1.281 | 0.0557† |  |  |
|  | 7.301 ± 0.660 |  | 6.281 ± 0.550 |  | 0.5473 to 1.492 | 1.674 | **0.0004**† |  |  |
|  | 7.301 ± 0.660 |  |  | 5.942 ± 0.359 | 0.8782 to 1.840 | 2.513 | **<0.0001**† |  |  |
|  |  | 6.580 ± 0.413 | 6.281 ± 0.550 |  | -0.2043 to 0.8023 | 0.609 | >0.9999† |  |  |
|  |  | 6.580 ± 0.413 |  | 5.942 ± 0.359 | 0.1271 to 1.149 | 1.653 | **0.0309**† |  |  |
|  |  |  | 6.281 ± 0.550 | 5.942 ± 0.359 | -0.1551 to 0.8334 | 0.752 | 0.6132† |  |  |
| **Level 5 (8.0 km/h, 18%)** |  | | | |  |  |  |  |  |
|  | 9.441 ± 0.539 | 8.839 ± 0.555 |  |  | -0.005189 to 1.209 | 1.099 | 0.2902† |  |  |
|  | 9.441 ± 0.539 |  | 8.371 ± 0.714 |  | 0.4722 to 1.668 | 1.664 | **0.0020**† |  |  |
|  | 9.441 ± 0.539 |  |  | 7.982 ± 0.482 | 0.8610 to 2.057 | 2.873 | **<0.0001**† |  |  |
|  |  | 8.839 ± 0.555 | 8.371 ± 0.714 |  | -0.1056 to 1.042 | 0.729 | 0.5870† |  |  |
|  |  | 8.839 ± 0.555 |  | 7.982 ± 0.482 | 0.2832 to 1.430 | 1.653 | **0.0118**† |  |  |
|  |  |  | 8.371 ± 0.714 | 7.982 ± 0.482 | -0.1748 to 0.9525 | 0.639 | 0.8551† |  |  |
| **Level 6 (8.8 km/h, 20%)** |  | | | |  |  |  |  |  |
|  | 11.438 | 10.616 ± 0.424 |  |  | -0.5929 to 2.238 | 1.939 | 0.4023 |  |  |
|  | 11.438 |  | 9.483 ± 0.195 |  | 0.4345 to 3.475 | 10.026 | **0.0079** |  |  |
|  | 11.438 |  |  | 9.756 ± 0.589 | 0.2775 to 3.086 | 2.856 | **0.0143** |  |  |
|  |  | 10.616 ± 0.424 | 9.483 ± 0.195 |  | 0.3473 to 1.917 | 2.931 | **0.0026** |  |  |
|  |  | 10.616 ± 0.424 |  | 9.756 ± 0.589 | 0.3328 to 1.386 | 1.872 | **0.0007** |  |  |
|  |  |  | 9.483 ± 0.195 | 9.756 ± 0.589 | -1.038 to 0.4923 | 0.505 | 0.7653 |  |  |
| **Level 7 (9.6 km/h, 22%)** |  | | | |  |  |  |  |  |
|  |  |  |  |  |  |  |  |  |  |
|  |  |  |  |  |  |  |  |  |  |
|  |  |  |  |  |  |  |  |  |  |
|  |  | 12.649 ± 0.013 | 11.302 ± 0.011 |  | -0.5365 to 3.230 | 111.862 | 0.1813 |  |  |
|  |  | 12.649 ± 0.013 |  | 11.643 ± 0.775 | -0.4327 to 2.444 | 1.356 | 0.1937 |  |  |
|  |  |  | 11.302 ± 0.011 | 11.643 ± 0.775 | -1.779 to 1.097 | 0.460 | 0.8087 |  |  |

Mechanic power output per skeletal muscle mass achieved by each group at each Bruce level as means ± SD. P-values, 95% confidence intervals of differences, and effect sizes through Hedges’ g are stated for differences between groups. † resembles results from non-parametric tests for non-normally distributed data. Statistically significant p-values printed in bold.

**Supplementary Table S12 RPE Values absolute (Multiple Comparison)**

| **Bruce Level** | **Women Untrained** | **Women Trained** | **Men Untrained** | **Men Trained** |  |  |  |  |  |
| --- | --- | --- | --- | --- | --- | --- | --- | --- | --- |
| **Level 0 (Rest)** |  | | | | 95% Confidence Interval of Differences | Hedges’ g | Multiple Comparison p-value |  |  |
|  | 0 ± 0 | 0 ± 0 |  |  | n/a | 0 | n/a |  |  |
|  | 0 ± 0 |  | 0 ± 0 |  | n/a | 0 | n/a |  |  |
|  | 0 ± 0 |  |  | 0 ± 0 | n/a | 0 | n/a |  |  |
|  |  | 0 ± 0 | 0 ± 0 |  | n/a | 0 | n/a |  |  |
|  |  | 0 ± 0 |  | 0 ± 0 | n/a | 0 | n/a |  |  |
|  |  |  | 0 ± 0 | 0 ± 0 | n/a | 0 | n/a |  |  |
| **Level 1 (2.7 km/h, 10%)** |  | | | |  |  |  |  |  |
|  | 6.412 ± 0.712 | 6.413 ± 0.363 |  |  | -0.3821 to 0.9200 | 0.002 | >0.9999† |  |  |
|  | 6.412 ± 0.712 |  | 6.938 ± 0.998 |  | -1.154 to 0.1026 | 0.613 | 0.3951† |  |  |
|  | 6.412 ± 0.712 |  |  | 6.143 ± 0.363 | -0.3821 to 0.9200 | 0.463 | >0.9999† |  |  |
|  |  | 6.413 ± 0.363 | 6.938 ± 0.998 |  | -1.455 to -0.1345 | 1.031 | **0.0384**† |  |  |
|  |  | 6.413 ± 0.363 |  | 6.143 ± 0.363 | -0.6818 to 0.6818 | 0.744 | >0.9999† |  |  |
|  |  |  | 6.938 ± 0.998 | 6.143 ± 0.363 | 0.1345 to 1.455 | 1.045 | **0.384**† |  |  |
| **Level 2 (4.0 km/h, 12%)** |  | | | |  |  |  |  |  |
|  | 7.941 ± 1.029 | 7.154 ± 1.068 |  |  | -0.5114 to 2.086 | 0.752 | 0.3840 |  |  |
|  | 7.941 ± 1.029 |  | 8.625 ± 1.857 |  | -1.912 to 0.5440 | 0.463 | 0.4593 |  |  |
|  | 7.941 ± 1.029 |  |  | 7.714 ± 1.138 | -1.045 to 1.499 | 0.210 | 0.9649 |  |  |
|  |  | 7.154 ± 1.068 | 8.625 ± 1.857 |  | -2.787 to -0.1550 | 0.954 | **0.0227** |  |  |
|  |  | 7.154 ± 1.068 |  | 7.714 ± 1.138 | -1.918 to 0.7972 | 0.507 | 0.6952 |  |  |
|  |  |  | 8.625 ± 1.857 | 7.714 ± 1.138 | -0.3793 to 2.201 | 0.587 | 0.2528 |  |  |
| **Level 3 (5.4 km/h, 14%)** |  | | | |  |  |  |  |  |
|  | 11.176 ± 1.944 | 9.429 ± 1.869 |  |  | -0.1395 to 3.635 | 0.786 | 0.0790 |  |  |
|  | 11.176 ± 1.944 |  | 10.875 ± 0.446 |  | -1.520 to 2.123 | 0.167 | 0.9716 |  |  |
|  | 11.176 ± 1.944 |  |  | 10.071 ± 1.439 | -0.7824 to 2.992 | 0.537 | 0.4154 |  |  |
|  |  | 9.429 ± 1.869 | 10.875 ± 0.446 |  | -3.360 to 0.4674 | 1.100 | 0.2000 |  |  |
|  |  | 9.429 ± 1.869 |  | 10.071 ± 1.439 | -2.619 to 1.334 | 0.387 | 0.8249 |  |  |
|  |  |  | 10.875 ± 0.446 | 10.071 ± 1.439 | -1.110 to 2.717 | 0.766 | 0.6842 |  |  |
| **Level 4 (6.7 km/h, 16%)** |  | | | |  |  |  |  |  |
|  | 14.353 ± 2.473 | 11.714 ± 1.634 |  |  | 0.8099 to 4.467 | 1.234 | **0.0014**† |  |  |
|  | 14.353 ± 2.473 |  | 13.75 ± 1.693 |  | -1.162 to 2.368 | 0.283 | >0.9999† |  |  |
|  | 14.353 ± 2.473 |  |  | 12.357 ± 1.598 | 0.1671 to 3.825 | 0.946 | **0.0261**† |  |  |
|  |  | 11.714 ± 1.634 | 13.75 ± 1.693 |  | -3.890 to -0.1814 | 1.222 | **0.0179**† |  |  |
|  |  | 11.714 ± 1.634 |  | 12.357 ± 1.598 | -2.558 to 1.272 | 0.398 | >0.9999† |  |  |
|  |  |  | 13.75 ± 1.693 | 12.357 ± 1.598 | -0.4615 to 3.247 | 0.845 | 0.1894† |  |  |
| **Level 5 (8.0 km/h, 18%)** |  | | | |  |  |  |  |  |
|  | 17.133 ± 1.922 | 14.214 ± 1.369 |  |  | 1.398 to 4.440 | 1.774 | **<0.0001** |  |  |
|  | 17.133 ± 1.922 |  | 16.5 ± 1.461 |  | -0.8379 to 2.105 | 0.377 | 0.6663 |  |  |
|  | 17.133 ± 1.922 |  |  | 14.357 ± 1.336 | 1.255 to 4.297 | 1.713 | **<0.0001** |  |  |
|  |  | 14.214 ± 1.369 | 16.5 ± 1.461 |  | -3.784 to -0.7876 | 1.613 | **0.0009** |  |  |
|  |  | 14.214 ± 1.369 |  | 14.357 ± 1.336 | -1.690 to 1.404 | 1.106 | 0.9948 |  |  |
|  |  |  | 16.5 ± 1.461 | 14.357 ± 1.336 | 0.6448 to 3.641 | 1.531 | **0.0021** |  |  |
| **Level 6 (8.8 km/h, 20%)** |  | | | |  |  |  |  |  |
|  | 18.000 | 16.357 ± 1.447 |  |  | -0.4941 to 3.780 | 1.135 | 0.1836 |  |  |
|  | 18.000 |  | 18.667 ± 1.073 |  | -2.843 to 1.509 | 0.622 | 0.8441 |  |  |
|  | 18.000 |  |  | 16.357 ± 1.598 | -0.4941 to 3.780 | 1.028 | 0.1836 |  |  |
|  |  | 16.357 ± 1.447 | 18.667 ± 1.073 |  | -3.792 to -0.8267 | 1.679 | **0.0009** |  |  |
|  |  | 16.357 ± 1.447 |  | 16.357 ± 1.598 | -1.425 to 1.425 | 0.000 | >0.9999 |  |  |
|  |  |  | 18.667 ± 1.073 | 16.357 ± 1.598 | 0.8267 to 3.792 | 1.660 | **0.0009** |  |  |
| **Level 7 (9.6 km/h, 22%)** |  | | | |  |  |  |  |  |
|  |  |  |  |  |  |  |  |  |  |
|  |  |  |  |  |  |  |  |  |  |
|  |  |  |  |  |  |  |  |  |  |
|  |  | 19.000 ± 0.414 | 19.500 ± 0.707 |  | -4.883 to 3.883 | 0.863 | >0.9999† |  |  |
|  |  | 19.000 ± 0.414 |  | 18.308 ± 1.750 | -2.637 to 4.022 | 0.412 | >0.9999† |  |  |
|  |  |  | 19.500 ± 0.707 | 18.308 ± 1.750 | -2.137 to 4.522 | 0.706 | 0.9648† |  |  |

RPE values absolute achieved by each group at each Bruce level as means ± SD. P-values, 95% confidence intervals of differences, and effect sizes through Hedges’ g are stated for differences between groups. † resembles results from non-parametric tests for non-normally distributed data. Statistically significant p-values printed in bold.
